# The scale of COVID‐19 graphs affects understanding, attitudes, and policy preferences

**DOI:** 10.1002/hec.4143

**Published:** 2020-08-25

**Authors:** Alessandro Romano, Chiara Sotis, Goran Dominioni, Sebastián Guidi

**Affiliations:** ^1^ Yale Law School New Haven Connecticut USA; ^2^ London School of Economics and Political Science London UK

**Keywords:** COVID‐19, framing, media, public understanding

## Abstract

Mass media routinely present data on coronavirus disease 2019 (COVID‐19) diffusion with graphs that use either a log scale or a linear scale. We show that the choice of the scale adopted on these graphs has important consequences on how people understand and react to the information conveyed. In particular, we find that when we show the number of COVID‐19 related deaths on a logarithmic scale, people have a less accurate understanding of how the pandemic has developed, make less accurate predictions on its evolution, and have different policy preferences than when they are exposed to a linear scale. Consequently, merely changing the scale the data is presented on can alter public policy preferences and the level of worry about the pandemic, despite the fact that people are routinely exposed to COVID‐19 related information. Providing the public with information in ways they understand better can help improving the response to COVID‐19, thus, mass media and policymakers communicating to the general public should always describe the evolution of the pandemic using a graph on a linear scale, at least as a default option. Our results suggest that framing matters when communicating to the public.

## 1 INTRODUCTION

The coronavirus disease 2019 (COVID‐19) pandemic is a formidable challenge. Absent a cure or a vaccine, it is crucial that people are adequately informed about the pandemic (Everett, Colombatto, Chituc, Brady, & Crockett, 2020), so that they stand behind policies that aim to minimize the spread of the virus and adopt behaviors that can limit the risk of contagion (Bursztyn, Rao, Roth, & Yanagizawa‐Drott, 2020). However, research has shown the challenges of communicating scientific facts in a way that effectively conveys essential information to the general public (Pidgeon & Fischhoff, 2011). In this article, we highlight the importance of this problem by focusing on one of the most basic pieces of information relative to the pandemic: the number of deaths.

To provide information on the diffusion of the virus, mass media routinely publish graphs that depict the evolution in the number of COVID‐19 related deaths in a given area. Many of these graphs present quantities on the Y‐axis on either a linear scale (The Washington Post, 2020; Vox, 2020) or a logarithmic scale (Financial Times, 2020; The Guardian, 2020; New York Times, 2020). The New York Times, for instance, has explained that the logarithmic scale helps better visualize exponential growth (New York Times, 2020). This follows advice given by epidemiology journals (Gladen, 1983; Levine, Ahmad, & Asa, 2010) and data visualization handbooks (Kosslyn, 2006). However, what might be true for conveying information among experts might not hold when issuing information to a broader audience. The principle that logarithmic scales are better suited for exponential growth does not hold true if readers do not, in fact, comprehend them.

We show that scale choice has important consequences on how people understand and react to the information conveyed. In particular, we find that when people are exposed to a logarithmic scale they have a less accurate understanding of how the pandemic unfolded until now, make less accurate predictions on its future, and have different attitudes and policy preferences than when they are exposed to a linear scale. Another study (Ryan & Evers, 2020) carried out a week after ours, confirms our finding that the scale of the graph affects policy preferences and that people have problems understanding logarithms. Instead, a study with Canadian respondents finds that the scale of the graph has no impact on respondents (Sevi et al., 2020).^1^ Previous studies have already shown that even experts have problems understanding graphs that use the logarithmic scale (Heckler, Mikula, & Rosenblatt, 2013; Menge et al., 2018). However, unlike most studies on graph comprehension we test understanding of graphs that represents real world highly salient data about which the public is likely to have ample background information and to care deeply. The obvious relevance of the data depicted in the graphs also allows us to test the impact of the scale in which the data is plotted on preferences about important policy issues. Since providing the public with clear information can help improving the response to COVID‐19 (Van Bavel et al., 2020), mass media and policymakers should present data on the evolution of the pandemic using a graph on a linear scale, at least as a default option.

## 2 EXPERIMENT

**FIGURE 1:**
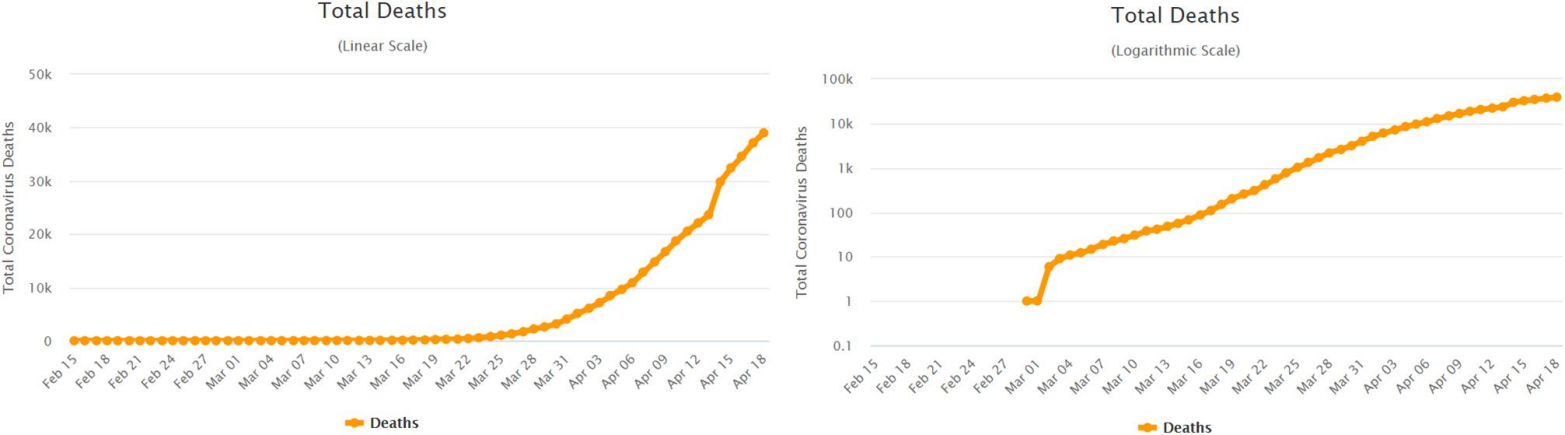
COVID‐19 related deaths in United States between February 15th and April 18th in a linear scale (left panel) and in a log scale (right panel). Source: www.worldometers.info

We devised a double‐blind experiment approved by the Yale IRB to test people's graph comprehension and its effects on attitudes and policy preferences. We recruited a sample of approximately *n* = 2000 (after exclusion criteria, with no regression with less than 1825 observations) U.S. residents on Cloud Research. Half of them were randomly assigned to the Linear Group, in which they were shown the evolution of COVID‐19 deaths in the U.S. on a linear scale. The other half were assigned to the Log Group, in which participants saw the same data, but plotted on a logarithmic scale. The graphs were taken from the popular website www.worldometers.info (see Figure 1). We asked respondents three sets of questions: (1) attitudes and policy preferences, (2) graph understanding, and (3) standard demographic questions. In the Appendix S1, we report the questions we asked and the order in which they were asked.

The analyses can be grouped into: (1) determinants of worry, (2) policy preferences, and (3) differences in understanding. In all three cases, our primary variable of interest is “linear,” a binary taking value 1 whenever the participant was exposed to the linear scale graphs, and 0 otherwise.

We start by showing participants in the two groups the graph plotting the evolution of the total number of deaths on the scale to which they were randomly assigned. Then we ask respondents in the two groups to indicate how worried they are about the health crisis and the economic crisis caused by COVID‐19 on a five points Likert scale from “not worried at all” to “extremely worried.” Second, we ask respondents about their preferences on some policies that many States have adopted to mitigate the spread of COVID‐19. In the first pair of policy questions we ask whether they support the policy of closing nonessential businesses (five points Likert scale from “strongly disagree” to “strongly agree”), and until which date they would keep these businesses closed. In the second pair of policy questions we ask participants how often they would use a mask if the government sent a supply (five points Likert scale from “never” to “always”). Moreover, we ask whether they would support a tax that finances the distribution of masks for everyone in their State (five points Likert scale from “strongly oppose” to “strongly support”).

We then turn to test respondents' understanding of the graphs. To increase external validity and to avoid priming respondents, we ask attitudes and policy preferences *before* testing understanding. This allows us to obtain respondents' policy preferences before they are asked to think thoroughly about the graph and its meaning in a way that they would be unlikely to do when reading actual news.

We test understanding of graphs by asking three questions. First, we show them the COVID‐19 graph on the scale that they had been assigned and ask them whether the number of deaths increased more between March 31st and April 6th or between April 6th and April 12th. Second, we show them a graph describing non‐COVID‐19 related data on the number of deaths from a hypothetical infection Z (taken from Okan, Galesic, & Garcia‐Retamero, 2016) and asked them a similar question. As for the first graph shown to participants, people in the Linear Group saw the data plotted on a linear scale, whereas respondents in the Log Group saw data plotted on a logarithmic one. The goal of this question was to test whether respondents' ability to answer correctly the first question depended on prior information on COVID‐19, or on a correct understanding of the scale on which their graphs are plotted.

Third, we test whether respondents can make predictions based on the curve. In particular, we ask them to make a prediction on the total number of deaths on April 25th, one week after we launched the experiment.

Predicting the number of COVID‐19 related deaths in a week is very difficult, but some predictions are more reasonable than others. We forecast the number of total deaths on April 25th using an ARIMA model, a standard forecasting method that has already been used to predict COVID‐19 diffusion (Benvenuto, Giovanetti, Vassallo, Angeletti, & Ciccozzi, 2020). We use an ARIMA (0,2,1), as simulations show that it offers the best fit for the data, and forecast the number of cases and its 95% and 99% confidence intervals (CIs). On the 18th of April the number of deaths was 39,014. The 95% CI forecasted using the ARIMA (0,2,1) ranges from 49,203.15 to 62,559.27, whereas the 99% CI ranges from 46,895.47 to 64,685.95. We remark that the actual number of deaths on the 25th of April was 54,256, while our ARIMA predicted 55,791 deaths predicted model. This is well within the CIs we consider.

We use these CIs to divide predictions in three groups. In the first group, we include the predictions that fall within the forecast 95% CI (“accurate range”). We consider these predictions “accurate.” In the second group, we include the predictions that fall within the 99% CI, but outside the 95% CI (“unlikely range”). We refer to these predictions as “unlikely.” Last, we consider the predictions that fall outside the 99% CI (“unreasonable range”) as “unreasonable.”

Additionally, for each of the understanding questions we asked how confident respondents were about their answers. The level of confidence is important as it can shed some light on how much weight people will attach to the information represented in the graph.

We concluded by collecting standard demographic information on the respondents.

## 3 RESULTS AND DISCUSSION

**FIGURE 2:**
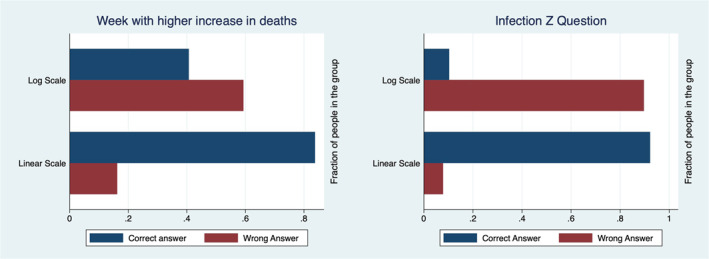
The left panel reports the percentage of correct and incorrect answers provided by the members of the two groups to the understanding question related to COVID‐19 real world data. The right panel reports the percentage of correct and incorrect answers provided by the members of the two groups to the understanding question related to Infection Z hypothetical data

**FIGURE 3:**
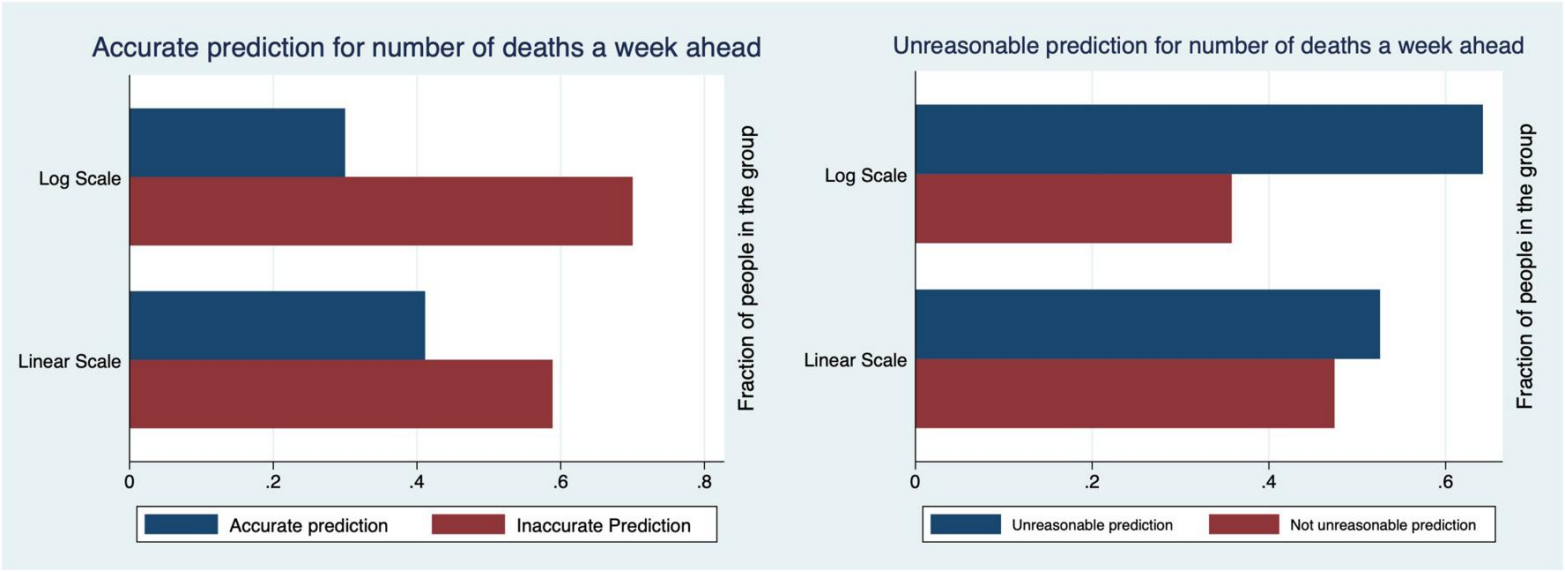
The left panel reports the percentage of accurate and inaccurate (i.e., not accurate) predictions provided by the members of the two groups. The right panel reports the unreasonable and reasonable (i.e., not unreasonable) predictions provided by the members of the two groups

Table 1 describes the characteristics of our sample. Figures 2 and 3 and Tables 2 and 3 show that people in the Linear Group understand the graphs better and make better predictions. The Log Group gives predictions that are higher and are on average unreasonable. Therefore, using linear scale graphs reduces the risk of confusing the public.

**TABLE 1:** Frequency table for demographic variables: Number, percentage, and cumulative percentage of respondents for the following variables: Age, education, income, political orientation, gender, live in city with less than 50K people, and live in city with more than 500K people

	Graph shown								
	Log scale			Linear scale			Total		
	No.	Percentage %	Cum %	No.	Percentage %	Cum %	No.	Percentage %	Cum %
Age									
18–25 years old	126	11.6	11.6	122	12.4	12.4	248	12.0	12.0
26–35 years old	351	32.3	43.9	309	31.3	43.7	660	31.8	43.8
36–45 years old	234	21.5	65.4	237	24.0	67.7	471	22.7	66.5
46–55 years old	182	16.7	82.2	150	15.2	82.9	332	16.0	82.5
56–65 years old	129	11.9	94.0	107	10.8	93.7	236	11.4	93.9
66–75 years old	57	5.2	99.3	52	5.3	99.0	109	5.3	99.1
>75 years old	8	0.7	100.0	10	1.0	100.0	18	0.9	100.0
Education									
Less than high school degree	4	0.4	0.4	5	0.5	0.5	9	0.4	0.4
High school graduate (diploma or equivalent)	88	8.1	8.5	83	8.4	8.9	171	8.3	8.7
Some college but no degree	210	19.3	27.8	168	17.0	26.0	378	18.2	26.9
Associate degree in college (2‐year)	97	8.9	36.7	101	10.2	36.2	198	9.6	36.5
Bachelor's degree in college	478	44.0	80.8	402	40.8	77.0	880	42.5	79.0
Master's degree or professional degree (JD, MD, etc)	190	17.5	98.3	203	20.6	97.6	393	19.0	97.9
Doctoral degree	19	1.7	100.0	24	2.4	100.0	43	2.1	100.0
Income									
Less than $10,000	48	4.4	4.4	36	3.7	3.7	84	4.1	4.1
$10,000–$19,999	64	5.9	10.3	56	5.7	9.3	120	5.8	9.9
$20,000–$29,999	75	6.9	17.2	96	9.8	19.1	171	8.3	18.1
$30,000–$39,999	120	11.1	28.3	88	8.9	28.0	208	10.1	28.2
$40,000–$49,999	108	10.0	38.2	104	10.6	38.6	212	10.2	38.4
$50,000–$59,999	111	10.2	48.5	103	10.5	49.1	214	10.3	48.8
$60,000–$69,999	100	9.2	57.7	85	8.6	57.7	185	8.9	57.7
$70,000–$79,999	100	9.2	66.9	75	7.6	65.3	175	8.5	66.2
$80,000–$89,999	58	5.3	72.3	68	6.9	72.3	126	6.1	72.3
$80,000–$89,999	60	5.5	77.8	71	7.2	79.5	131	6.3	78.6
$90,000–$99,999	164	15.1	92.9	128	13.0	92.5	292	14.1	92.7
$150,000 or more	77	7.1	100.0	74	7.5	100.0	151	7.3	100.0
Political orientation									
Other	352	32.4	32.4	292	29.6	29.6	644	31.1	31.1
Democrat	441	40.6	73.0	426	43.2	72.7	867	41.8	72.9
Republican	294	27.0	100.0	269	27.3	100.0	563	27.1	100.0
Total	1087	100.0		987	100.0		2074	100.0	
Gender									
Other/prefer not to declare	8	0.7	0.7	14	1.4	1.4	22	1.1	1.1
Female	571	52.5	53.3	524	53.1	54.5	1095	52.8	53.9
Male	508	46.7	100.0	449	45.5	100.0	957	46.1	100.0
Live in city with <50K people									
No	680	62.6	62.6	601	60.9	60.9	1281	61.8	61.8
Yes	407	37.4	100.0	386	39.1	100.0	793	38.2	100.0
Total	1087	100.0		987	100.0		2074	100.0	
Live in city with >500K people									
No	851	78.3	78.3	769	77.9	77.9	1620	78.1	78.1
Yes	236	21.7	100.0	218	22.1	100.0	454	21.9	100.0

*Note*: Column 1 shows overall distribution, Column 2 shows the distribution for the Linear Group, and Column 3 shows the one for the Log Group.

**TABLE 2:** Understanding questions: The coefficients are estimated through a Logit regression

	Understanding Q.1: Real data		Understanding Q.2: Hypothetical	
	(1)	(2)	(3)	(4)
In Linear Group	2.021*** (0.000)	2.054*** (0.000)	4.634*** (0.000)	4.819*** (0.000)
Confidence in understanding Q.1		0.00886*** (0.000)		
Worry about health crisis		−0.0310 (0.585)		−0.0851 (0.318)
COVID‐19 news checking		0.0780 (0.145)		0.0860 (0.290)
Education		0.0213 (0.619)		0.152** (0.021)
Male		−0.147 (0.193)		0.321* (0.066)
Age		0.00445 (0.268)		0.0154** (0.012)
Democrat		0.00380 (0.977)		0.0870 (0.660)
Republican		−0.0190 (0.895)		−0.183 (0.413)
Confidence in understanding Q.2				0.0308*** (0.000)
Constant	−0.378*** (0.000)	−1.375*** (0.001)	−2.164*** (0.000)	−6.119*** (0.000)
Observations	2074	1830	2074	1830

*Note*: *p*‐values are reported in parentheses. The standard errors can be found in the Appendix S1. Columns 1 and 2: Right answer to the question on the understanding question on COVID‐19 data. Columns 3 and 4: Right answer to question on Infection Z (hypothetical data). All coefficients for the control variables are reported.

*p < 0.10, **p < 0.05, ***p < 0.01.

**TABLE 3:** Determinants of making an accurate prediction (Columns 1 and 2) and an unreasonable prediction (Columns 3 and 4)

	Accurate prediction		Unreasonable prediction	
	(1)	(2)	(3)	(4)
In Linear Group	0.489*** (0.000)	0.482*** (0.000)	−0.481*** (0.000)	−0.480*** (0.000)
Confidence in prediction		−0.00178 (0.447)		0.00188 (0.411)
Worry about health crisis		−0.0112 (0.830)		0.0494 (0.327)
COVID‐19 news checking		0.150*** (0.002)		−0.175*** (0.000)
Education		0.0477 (0.221)		−0.0461 (0.224)
Male		−0.0327 (0.749)		−0.0149 (0.881)
Age		0.00182 (0.616)		−0.00480 (0.175)
Democrat		0.0920 (0.437)		−0.106 (0.360)
Republican		−0.181 (0.172)		0.221* (0.087)
Constant	−0.848*** (0.000)	−1.378*** (0.000)	0.585*** (0.000)	1.147*** (0.001)
Observations	2074	1832	2074	1832

*Note*: The coefficients are estimated through Logit regressions. *p*‐values are reported in parentheses. The standard errors can be found in the Appendix S1. All coefficients for the control variables are reported.

*p < 0.10, ***p < 0.01.

Moreover, the scale also impacts people level of worry for the health crisis (but not for the economic crisis) and their policy preferences. People in the Linear Group are more worried about the health crisis (see Table 4), and prefer that nonessential businesses remain closed for longer (Table 5). However, they support less strongly the idea of closing nonessential business in the first place (Table 5), and would wear government‐supplied masks less often (Table 6). These results are statistically significant and robust to a series of different controls and specifications (the regressions presented use Logit and OLS and the results are robust to different sets of controls). The odds ratios show that the magnitude of the effects is non‐negligible (Table 7).

**TABLE 4:** Determinants of worry about health crisis caused by COVID‐19

	Worry about health crisis		
	(1)	(2)	(3)
In Linear Group	0.141* (0.081)	0.258* (0.091)	0.327** (0.038)
COVID‐19 news checking		0.500*** (0.000)	0.434*** (0.000)
Male		−0.806*** (0.000)	−0.654*** (0.000)
Understanding Q.1: Real data		−0.00425 (0.967)	0.00558 (0.958)
Confidence in understanding Q.1		−0.00134 (0.706)	−0.00152 (0.674)
Understanding Q.2: Hypothetical		−0.137 (0.386)	−0.225 (0.171)
Confidence in understanding Q.2		−0.00374 (0.302)	−0.00428 (0.246)
Accurate prediction		0.156 (0.404)	0.218 (0.255)
Unreasonable prediction		0.225 (0.216)	0.325* (0.084)
Confidence in prediction		0.00622*** (0.005)	0.00579*** (0.009)
Democrat			0.732*** (0.000)
Republican			−0.282** (0.017)
Worry about economic crisis			0.707*** (0.000)
Live in city with <50K people			0.0156 (0.880)
Live in city with >500K people			−0.132 (0.280)
Education			−0.0258 (0.473)
Age			−0.00132 (0.694)
State of residence			0.00777** (0.030)
Restrictions in the state			−0.156 (0.160)
Observations	2074	1837	1828

*Note*: The coefficients are estimated through ordered Logit regressions. *p*‐values are reported in parentheses. Standard errors can be found in the Appendix S1. All coefficients for the control variables are reported.

*p < 0.10, **p < 0.05, ***p < 0.01.

**TABLE 5:** Determinants for support for keeping shops closed (Columns 1–3) and suggested reopening day (Columns 4–6)

	Support for closing businesses			Days until reopening businesses		
	(1)	(2)	(3)	(4)	(5)	(6)
In Linear Group	0.0406 (0.621)	−0.378** (0.019)	−0.424** (0.012)	2.295 (0.464)	17.38** (0.014)	14.65** (0.037)
Worry about health crisis		0.997*** (0.000)	1.067*** (0.000)		12.45*** (0.000)	13.14*** (0.000)
COVID‐19 news checking		0.0288 (0.531)	0.0748 (0.117)		3.071* (0.056)	3.932** (0.018)
Male		−0.112 (0.242)	−0.0890 (0.366)		10.53*** (0.002)	9.169*** (0.006)
Understanding Q.1: Real data		0.131 (0.228)	0.132 (0.236)		−1.236 (0.762)	−0.517 (0.900)
Confidence in understanding Q.1		0.00955*** (0.009)	0.00842** (0.023)		0.109 (0.391)	0.0996 (0.440)
Understanding Q.2: Hypothetical		0.300* (0.075)	0.348** (0.047)		−18.05** (0.012)	−15.87** (0.026)
Confidence in understanding Q.2		−0.000421 (0.911)	−0.000228 (0.952)		−0.310** (0.025)	−0.299** (0.032)
Accurate prediction		0.480** (0.012)	0.450** (0.019)		10.58* (0.093)	9.343 (0.138)
Unreasonable prediction		0.0871 (0.635)	0.0806 (0.665)		6.590 (0.277)	4.787 (0.431)
Confidence in prediction		−0.00451* (0.054)	−0.00426* (0.073)		0.216*** (0.007)	0.205** (0.012)
Democrat			0.545*** (0.000)			0.107 (0.977)
Republican			−0.491*** (0.000)			1.912 (0.683)
Worry about economic crisis			−0.494*** (0.000)			−3.597* (0.069)
Live in city with <50K people			0.0314 (0.770)			6.259* (0.085)
Live in city with >500K people			0.0230 (0.858)			9.164** (0.037)
Education			−0.0258 (0.496)			−1.798 (0.173)
Age			−0.00105 (0.769)			−0.151 (0.192)
State of residence			0.00274 (0.456)			−0.00686 (0.957)
Restrictions in the state			−0.0175 (0.881)			−1.382 (0.741)
Constant				65.38*** (0.000)	−0.312 (0.979)	24.09 (0.155)
Observations	2074	1837	1828	2061	1828	1819

*Note*: Columns 1–3 report coefficients estimated through ordered Logit regressions and Columns 4–6 report coefficients obtained through ordinary least squares regressions (OLS). *p*‐values are reported in parentheses. The standard errors can be found in the Appendix S1. All coefficients for the control variables are reported table.

*p < 0.10, **p < 0.05, ***p < 0.01.

**TABLE 6:** Determinants of likelihood to wear a mask when going out if provided with one (Columns 1–3) and supporting a tax to finance their distribution (Columns 4–6)

	Likelihood to wear masks			Support for mask‐buying tax		
	(1)	(2)	(3)	(4)	(5)	(6)
In Linear Group	0.00311 (0.970)	−0.314** (0.045)	−0.350** (0.029)	−0.0218 (0.780)	0.307** (0.042)	0.305** (0.046)
Worry about health crisis		0.907*** (0.000)	0.908*** (0.000)		0.481*** (0.000)	0.471*** (0.000)
COVID‐19 news checking		0.138*** (0.003)	0.129*** (0.006)		0.0403 (0.341)	0.0682 (0.116)
Male		−0.255*** (0.007)	−0.270*** (0.005)		0.0372 (0.673)	0.0455 (0.612)
Understanding Q.1: Real data		0.0281 (0.796)	0.0136 (0.902)		0.152 (0.133)	0.169* (0.097)
Confidence in understanding Q.1		0.00571 (0.125)	0.00493 (0.192)		0.00648* (0.065)	0.00602* (0.088)
Understanding Q.2: Hypothetical		0.189 (0.249)	0.237 (0.157)		−0.454*** (0.004)	−0.452*** (0.004)
Confidence in understanding Q.2		0.00250 (0.510)	0.00272 (0.479)		−0.0108*** (0.003)	−0.0112*** (0.002)
Accurate prediction		0.435** (0.020)	0.431** (0.022)		0.186 (0.312)	0.141 (0.444)
Unreasonable prediction		0.497*** (0.007)	0.493*** (0.007)		0.165 (0.357)	0.147 (0.414)
Confidence in prediction		0.00211 (0.352)	0.00276 (0.231)		0.00675*** (0.002)	0.00734*** (0.001)
Democrat			0.161 (0.154)			0.378*** (0.000)
Republican			−0.384*** (0.001)			−0.261** (0.024)
Worry about economic crisis			−0.132** (0.021)			−0.0979* (0.069)
Live in city with <50K people			0.0832 (0.424)			0.115 (0.240)
Live in city with >500K people			0.588*** (0.000)			0.0488 (0.681)
Education			−0.0767** (0.040)			−0.0209 (0.543)
Age			0.00713** (0.041)			−0.00942*** (0.004)
State of residence			0.0170*** (0.000)			−0.00313 (0.358)
Restrictions in the state			−0.154 (0.177)			−0.122 (0.258)
Likelihood to wear masks					0.648*** (0.000)	0.617*** (0.000)
Observations	2072	1835	1826	2072	1834	1825

*Note*: The coefficients are estimated through ordered Logit regressions. *p*‐values are reported in parentheses. The standard errors can be found in the Appendix S1. All coefficients for the control variables are reported table.

*p < 0.10, **p < 0.05, ***p < 0.01.

**TABLE 7:** The table reports odds ratios for Logit regressions: Worry about health crisis, likelihood to wear masks, support for mask‐buying tax, support for closing businesses, understanding Q.1: Real data, understanding Q.2: Hypothetical, accurate prediction, unreasonable prediction

	Worry about health crisis	Likelihood to wear masks	Support for mask‐buying tax	Support for closing businesses	Understanding Q.1: Real data	Understanding Q.2: Hypothetical	Accurate prediction	Unreasonable prediction
	(1)	(2)	(3)	(4)	(5)	(6)	(7)	(8)
In Linear Group	1.387* (0.218)	0.705* (0.113)	1.356* (0.207)	0.654* (0.110)	7.800*** (0.902)	123.9*** (23.13)	1.619*** (0.159)	0.619*** (0.0594)
COVID‐19 news checking	1.543*** (0.0718)	1.138** (0.0537)	1.071 (0.0464)	1.078 (0.0514)	1.081 (0.0578)	1.090 (0.0886)	1.162** (0.0563)	0.840*** (0.0398)
Male	0.520*** (0.0486)	0.763** (0.0735)	1.047 (0.0937)	0.915 (0.0900)	0.864 (0.0972)	1.379 (0.241)	0.968 (0.0988)	0.985 (0.0980)
Understanding Q.1: Real data	1.006 (0.107)	1.014 (0.112)	1.184 (0.120)	1.141 (0.127)				
Confidence in understanding Q.1	0.998 (0.00361)	1.005 (0.00379)	1.006 (0.00355)	1.008* (0.00375)	1.009*** (0.00253)			
Understanding Q.2: Hypothetical	0.799 (0.131)	1.267 (0.212)	0.636** (0.101)	1.416* (0.247)				
Confidence in understanding Q.2	0.996 (0.00368)	1.003 (0.00385)	0.989 (0.00360)	1.000 (0.00379)		1.031*** (0.00424)		
Accurate prediction	1.244 (0.238)	1.539* (0.290)	1.152 (0.213)	1.569* (0.302)				
Unreasonable prediction	1.384 (0.260)	1.638** (0.301)	1.159 (0.209)	1.084 (0.202)				
Confidence in prediction	1.006** (0.00225)	1.003 (0.00231)	1.007*** (0.00221)	0.996 (0.00236)			0.998 (0.00234)	1.002 (0.00229)
Democrat	2.080*** (0.225)	1.175 (0.133)	1.459*** (0.152)	1.725*** (0.200)	1.004 (0.131)	1.091 (0.216)	1.096 (0.130)	0.900 (0.104)
Republican	0.754* (0.0893)	0.681** (0.0822)	0.770* (0.0891)	0.612*** (0.0735)	0.981 (0.141)	0.833 (0.186)	0.834 (0.111)	1.247 (0.161)
Worry about economic crisis	2.028*** (0.112)	0.876* (0.0502)	0.907 (0.0488)	0.610*** (0.0374)				
Live in city with ¡50K people	1.016 (0.105)	1.087 (0.113)	1.122 (0.110)	1.032 (0.111)				
Live in city with ¿500K people	0.876 (0.107)	1.801*** (0.233)	1.050 (0.124)	1.023 (0.132)				
Education	0.975 (0.0350)	0.926* (0.0347)	0.979 (0.0338)	0.975 (0.0369)	1.022 (0.0438)	1.164* (0.0768)	1.049 (0.0409)	0.955 (0.0362)
Age	0.999 (0.00336)	1.007* (0.00352)	0.991** (0.00322)	0.999 (0.00355)	1.004 (0.00403)	1.016* (0.00624)	1.002 (0.00363)	0.995 (0.00352)
State of residence	1.008* (0.00362)	1.017*** (0.00402)	0.997 (0.00339)	1.003 (0.00368)				
Restrictions in the state	0.855 (0.0951)	0.857 (0.0978)	0.885 (0.0957)	0.983 (0.115)				
Worry about health crisis		2.480*** (0.136)	1.602*** (0.0862)	2.907*** (0.165)	0.969 (0.0550)	0.918 (0.0782)	0.989 (0.0513)	1.051 (0.0530)
Likelihood to wear masks			1.854*** (0.0935)					
Observations	1828	1826	1825	1828	1830	1830	1832	1832

*Note*: The controls used in each of these regression are the same as in the last column of each regression in Tables 2, 3, 4, 5, 6. Exponentiated coefficients; Standard errors in parentheses.

*p < 0.05, **p < 0.01, ***p < 0.001.

These findings are remarkable because the data underlying the graphs is identical. Merely changing the scale can alter public policy preferences and the level of worry, despite the endless flow of COVID‐19 related information to which everyone is exposed.

We cannot know the mechanism leading to these preferences, but we advance the conjecture that the shape of the curves could explain these findings. The flat logarithmic curve can give the impression that we reached a plateau and that, while the present situation is very serious, things are about to get better soon. Thus respondents in the Log Group might be less worried because they feel that the end of the pandemic is near. For the same reason, they could strongly support closing nonessential businesses now, that is, during the peak, but could want to reopen them as soon as the peak is over. Moreover, they might concentrate the use of masks during the peak. As the Log Group thinks we are at the peak, they could also expect a very high number of deaths in the short term, which would also explain their strong support to wear masks and to keep business closed.

Vice versa, the linear curve is constantly growing with no sign of improvement, hence it might give the impression that the crisis will go on for long and will be very serious. Consequently, people in the Linear Group might be more worried and wish to reopen nonessential businesses later. However, they could support closing nonessential businesses relatively less, because they believe that the pandemic will last for a long time, and nonessential businesses cannot remain closed for too long. However, if the decision taken is to close nonessential businesses, they might feel that it would be pointless to do it for a short period of time. They would apply a similar logic to masks. As they believe that the pandemic will last for a long time, they could use them less frequently to ration them.

Regardless of the reasons behind our findings, it is noteworthy that changing the scale can alter policy preferences, intentions to adopt precautionary measures, and level of worry for the health consequences of the pandemic. Given that the scale affects policy preferences and that people have significant problems understanding the logarithmic scale, our findings suggest that representing data on a linear scale is preferable. Garfin, Silver, and Holman (2020) noted that during a public health crisis, the general public relies on the media to convey accurate and understandable information, so that it can take informed decisions regarding health protective behaviors. Absent information of this kind, people cannot form informed preferences or take informed decisions. Moreover, unclear information conveyed by the media could undermine how much people trust science, which is a key predictor of compliance with COVID‐19 guidelines (Brzezinski, Kecht, Van Dijcke, & Wright Austin, 2020; Phlol & Musil, 2020).

## Supporting information

Supplementary Material

Supplementary Material

Supplementary Material

## ENDNOTE

^1^
However, their study uses a “catch all” question for pessimism and one on policy preferences. These catch all questions might be unable to capture the nuanced impact of graph scale on policies and attitudes that we observe. For instance, we observe an impact on worry for the health crisis, but *not* on worry for the economic crisis.
