# Supplementary material for: The scale of COVID‐19 graphs affects understanding, attitudes, and policy preferences
Source: Health Econ. 2020 Aug 25;29(11):1482–94. doi: 10.1002/hec.4143 (PMC7461444; doi:10.1002/hec.4143)
Supplement: Supplementary file 1 — Supplementary Material [file HEC-29-1482-s002.pdf]

Logarithmic COVID19

---

1. Appendix

In the appendix we include additional tables that expand on the results presented in the article and the full questionnaire.

Table A1: Summary statistics (mean and standard deviation (SD)) for the variables considered in the regression tables. These include: Worry About Health Crisis, Worry About Economic Crisis, Days Until Reopening Businesses, Support for Closing Businesses, Likelihood to Wear Masks and Support for a Mask-Buying Tax. Column 1 presents the statistics for all participants, Column 2 only for the Linear Group and Column 3 only for the Log Group.

	All Sample		Linear Group		Log Group	
	mean	SD	mean	SD	mean	SD
Worry About Health Crisis	3.98	1.07	4.03	1.05	3.94	1.10
Worry About Economic Crisis	4.25	0.90	4.27	0.86	4.23	0.93
Days Until Reopening Businesses	66.47	71.01	67.67	71.19	65.38	70.87
Support for Closing Businesses	4.08	1.16	4.09	1.16	4.07	1.17
Likelihood to Wear Masks	4.09	1.10	4.10	1.09	4.09	1.10
Support for Mask-Buying Tax	3.25	1.38	3.24	1.38	3.25	1.39
Observations	2074		987		1087	

Table A2: Determinants of answering the understanding question on COVID-19 data (Columns 1-4) and the understanding question on Infection Z (hypothetical data) (Columns 5-8) correctly. Columns 1, 2, 5 and 6 report coefficients estimated through Logit regressions, Columns 3, 4, 7 and 8 report coefficients estimated through Probit regressions. Standard errors are reported in parentheses. All coefficients for the control variables are reported.

	(1)<br>Understanding Q.1:<br>Real Data	(2)<br>Understanding Q.1:<br>Real Data	(3)<br>Understanding Q.1:<br>Real Data	(4)<br>Understanding Q.1:<br>Real Data	(5)<br>Understanding Q.2:<br>Hypothetical	(6)<br>Understanding Q.2:<br>Hypothetical	(7)<br>Understanding Q.2:<br>Hypothetical	(8)<br>Understanding Q.2:<br>Hypothetical
In Linear Group	2.021***	2.054***	1.222***	1.241***	4.634***	4.819***	2.683***	2.733***
	(0.106)	(0.116)	(0.0613)	(0.0664)	(0.155)	(0.187)	(0.0779)	(0.0899)
Confidence in Understanding Q.1		0.00886***		0.00560***				
		(0.00251)		(0.00147)				
Worry About Health Crisis		-0.0310		-0.0201		-0.0851		-0.0492
		(0.0568)		(0.0339)		(0.0852)		(0.0450)
COVID-19 News Checking		0.0780		0.0430		0.0860		0.0457
		(0.0535)		(0.0317)		(0.0813)		(0.0420)
Education		0.0213		0.0152		0.152**		0.0795**
		(0.0429)		(0.0254)		(0.0659)		(0.0341)
Male		-0.147		-0.0875		0.321*		0.160*
		(0.113)		(0.0670)		(0.175)		(0.0895)
Age		0.00445		0.00261		0.0154**		0.00748**
		(0.00401)		(0.00239)		(0.00614)		(0.00317)
Democrat		0.00380		-0.00114		0.0870		0.0302
		(0.130)		(0.0778)		(0.198)		(0.103)
Republican		-0.0190		-0.0140		-0.183		-0.0992
		(0.144)		(0.0856)		(0.223)		(0.115)
Confidence in Understanding Q.2						0.0308***		0.0148***
						(0.00411)		(0.00204)
Constant	-0.378***	-1.375***	-0.236***	-0.843***	-2.164***	-6.119***	-1.264***	-3.136***
	(0.0617)	(0.407)	(0.0384)	(0.240)	(0.0998)	(0.665)	(0.0514)	(0.334)
Observations	2074	1830	2074	1830	2074	1830	2074	1830
Adjusted R^2								

Standard errors in parentheses
* $p < 0.10$, ** $p < 0.05$, *** $p < 0.01$

Table A3: Determinants of making an accurate prediction (Columns 1-4) and an unreasonable prediction (Columns 5-8). The coefficients estimated through Logit regressions (Columns 1, 2, 5 and 6) and a Probit regressions (Columns 3, 4, 7 and 8). Standard errors in parentheses. All coefficients for the control variables are reported.

	(1) Accurate Prediction	(2) Accurate Prediction	(3) Accurate Prediction	(4) Accurate Prediction	(5) Unreasonable Prediction	(6) Unreasonable Prediction	(7) Unreasonable Prediction	(8) Unreasonable Prediction
Prediction								
In Linear Group	0.489***	0.482***	0.301***	0.297***	-0.481***	-0.480***	-0.299***	-0.299***
	(0.0926)	(0.0985)	(0.0567)	(0.0605)	(0.0898)	(0.0961)	(0.0558)	(0.0596)
Confidence in Prediction		-0.00178		-0.00110		0.00188		0.00117
		(0.00234)		(0.00144)		(0.00228)		(0.00142)
Worry About Health Crisis		-0.0112		-0.00652		0.0494		0.0300
		(0.0519)		(0.0318)		(0.0504)		(0.0313)
COVID-19 News Checking		0.150***		0.0931***		-0.175***		-0.109***
		(0.0484)		(0.0298)		(0.0474)		(0.0294)
Education		0.0477		0.0295		-0.0461		-0.0288
		(0.0390)		(0.0239)		(0.0379)		(0.0236)
Male		-0.0327		-0.0201		-0.0149		-0.00876
		(0.102)		(0.0627)		(0.0995)		(0.0617)
Age		0.00182		0.00113		-0.00480		-0.00300
		(0.00363)		(0.00223)		(0.00354)		(0.00220)
Democrat		0.0920		0.0573		-0.106		-0.0657
		(0.118)		(0.0728)		(0.116)		(0.0718)
Republican		-0.181		-0.110		0.221*		0.137*
		(0.133)		(0.0812)		(0.129)		(0.0797)
Constant	-0.848***	-1.378***	-0.525***	-0.857***	0.585***	1.147***	0.364***	0.719***
	(0.0662)	(0.346)	(0.0400)	(0.212)	(0.0633)	(0.337)	(0.0389)	(0.209)
Observations	2074	1832	2074	1832	2074	1832	2074	1832
Adjusted R^2								

Standard errors in parentheses
* $p < 0.10$, ** $p < 0.05$, *** $p < 0.01$

Table A4: Determinants of worry about the economic crisis caused by Covid-19. The coefficients are estimated through ordered Logit regressions (Columns 1-3), ordered Probit regressions (Columns 4-6) and Ordinary Least Squares (OLS) regressions (Columns 7-9). Standard errors are reported in parentheses. All coefficients for the control variables are reported.

	(1) Worry About Economic Crisis	(2) Worry About Economic Crisis	(3) Worry About Economic Crisis	(4) Worry About Economic Crisis	(5) Worry About Economic Crisis	(6) Worry About Economic Crisis	(7) Worry About Economic Crisis	(8) Worry About Economic Crisis	(9) Worry About Economic Crisis
main									
In Linear Group	0.0397	-0.116	-0.102	0.0372	-0.0387	-0.0331	0.0384	-0.0200	-0.0159
	(0.0828)	(0.161)	(0.163)	(0.0494)	(0.0928)	(0.0939)	(0.0393)	(0.0727)	(0.0733)
Worry About Health Crisis		0.535***	0.598***		0.303***	0.341***		0.227***	0.250***
		(0.0492)	(0.0513)		(0.0274)	(0.0284)		(0.0256)	(0.0260)
COVID-19 News Checking		0.223***	0.214***		0.127***	0.119***		0.0822***	0.0764***
		(0.0453)	(0.0464)		(0.0267)	(0.0273)		(0.0184)	(0.0188)
Male		-0.0695	-0.0852		-0.0604	-0.0705		-0.0476	-0.0554
		(0.0943)	(0.0958)		(0.0554)	(0.0562)		(0.0405)	(0.0407)
Understanding Q.1: Real Data		-0.107	-0.116		-0.0802	-0.0844		-0.0652	-0.0677
		(0.108)	(0.109)		(0.0634)	(0.0638)		(0.0456)	(0.0456)
Confidence in Understanding Q.1		-0.0000464	0.000460		-0.0000710	0.000326		-0.000215	-0.0000115
		(0.00365)	(0.00369)		(0.00214)	(0.00216)		(0.00155)	(0.00156)
Understanding Q.2: Hypothetical		0.174	0.170		0.0950	0.0934		0.0685	0.0675
		(0.168)	(0.170)		(0.0972)	(0.0982)		(0.0740)	(0.0745)
Confidence in Understanding Q.2		-0.000656	-0.000441		-0.000524	-0.000551		-0.000289	-0.000261
		(0.00375)	(0.00378)		(0.00219)	(0.00220)		(0.00158)	(0.00161)
Accurate Prediction		-0.304	-0.289		-0.181	-0.172		-0.130*	-0.125
		(0.197)	(0.198)		(0.116)	(0.116)		(0.0763)	(0.0761)
Unreasonable Prediction		-0.256	-0.269		-0.163	-0.169		-0.125*	-0.128*
		(0.193)	(0.194)		(0.113)	(0.113)		(0.0748)	(0.0748)
Confidence in Prediction		0.000260	0.000311		-0.0000446	0.0000609		-0.000353	-0.000269
		(0.00226)	(0.00229)		(0.00133)	(0.00135)		(0.00100)	(0.00100)
Democrat			-0.294***			-0.175***			-0.128***
			(0.110)			(0.0649)			(0.0470)
Republican			0.231*			0.125*			0.0740
			(0.124)			(0.0724)			(0.0517)
Live in city with <50K People			-0.147			-0.0862			-0.0498
			(0.104)			(0.0614)			(0.0444)
Live in city with >500K People			0.0144			0.0227			0.0230
			(0.125)			(0.0735)			(0.0518)
Education			-0.0174			-0.00736			-0.00358
			(0.0367)			(0.0215)			(0.0153)
Age			0.00481			0.00309			0.00196
			(0.00343)			(0.00202)			(0.00139)
State of Residence			-0.000372			-0.000867			-0.000649
			(0.00353)			(0.00207)			(0.00160)
Restrictions in the State			0.233**			0.133*			0.0916**
			(0.115)			(0.0683)			(0.0465)
Constant							4.231***	3.321***	3.033***
							(0.0281)	(0.151)	(0.203)
Observations	2073	1837	1828	2073	1837	1828	2073	1837	1828
Adjusted R^2							-0.000	0.092	0.102

Standard errors in parentheses
* $p < 0.10$, ** $p < 0.05$, *** $p < 0.01$

Table A5: Determinants of worry about health crisis caused by COVID-19. The coefficients are estimated through ordered Logit regressions (Columns 1-3), ordered Probit regressions (Columns 4-6) and Ordinary Least Squares (OLS) regressions (Columns 7-9). Standard errors are reported in parentheses. All coefficients for the control variables are reported.

	(1)<br>Worry About Health<br>Health Crisis	(2)<br>Worry About<br>Health Crisis	(3)<br>Worry About<br>Health Crisis	(4)<br>Worry About<br>Health Crisis	(5)<br>Worry About<br>Health Crisis	(6)<br>Worry About<br>Health Crisis	(7)<br>Worry About<br>Health Crisis	(8)<br>Worry About<br>Health Crisis	(9)<br>Worry About Crisis<br>Health Crisis
In Linear Group	0.141*	0.258*	0.327**	0.0833*	0.161*	0.161*	0.0900*	0.135*	0.130*
	(0.0806)	(0.153)	(0.157)	(0.0478)	(0.0905)	(0.0920)	(0.0470)	(0.0697)	(0.0695)
COVID-19 News Checking		0.500***	0.434***		0.243***	0.239***		0.179***	0.170***
		(0.0442)	(0.0465)		(0.0260)	(0.0267)		(0.0217)	(0.0217)
Male		-0.806***	-0.654***		-0.437***	-0.382***		-0.362***	-0.308***
		(0.0906)	(0.0934)		(0.0535)	(0.0545)		(0.0442)	(0.0434)
Understanding Q.1: Real Data		-0.00425	0.00558		0.0102	0.00885		0.00256	0.00592
		(0.104)	(0.107)		(0.0619)	(0.0625)		(0.0509)	(0.0501)
Confidence in Understanding Q.1		-0.00134	-0.00152		-0.000494	-0.000967		-0.000548	-0.000952
		(0.00356)	(0.00362)		(0.00209)	(0.00210)		(0.00175)	(0.00177)
Understanding Q.2: Hypothetical		-0.137	-0.225		-0.0989	-0.108		-0.0737	-0.0822
		(0.158)	(0.164)		(0.0945)	(0.0959)		(0.0729)	(0.0731)
Confidence in Understanding Q.2		-0.00374	-0.00428		-0.00239	-0.00240		-0.00233	-0.00225
		(0.00362)	(0.00369)		(0.00213)	(0.00215)		(0.00170)	(0.00172)
Accurate Prediction		0.156	0.218		0.155	0.155		0.139	0.134
		(0.186)	(0.192)		(0.109)	(0.110)		(0.102)	(0.0990)
Unreasonable Prediction		0.225	0.325*		0.182*	0.206*		0.143	0.154
		(0.182)	(0.188)		(0.107)	(0.107)		(0.100)	(0.0974)
Confidence in Prediction		0.00622***	0.00579***		0.00322**	0.00304**		0.00226**	0.00207*
		(0.00219)	(0.00223)		(0.00129)	(0.00131)		(0.00110)	(0.00108)
Democrat			0.732***			0.448***			0.356***
			(0.108)			(0.0633)			(0.0505)
Republican			-0.282**			-0.154**			-0.154**
			(0.118)			(0.0685)			(0.0638)
Worry About Economic Crisis			0.707***		0.352***	0.380***		0.295***	0.309***
			(0.0555)		(0.0303)	(0.0307)		(0.0314)	(0.0302)
Live in city with <50K People			0.0156			0.0222			0.0255
			(0.103)			(0.0599)			(0.0495)
Live in city with >500K People			-0.132			-0.0588			-0.0538
			(0.122)			(0.0716)			(0.0586)
Education			-0.0258			-0.0213			-0.0128
			(0.0359)			(0.0210)			(0.0171)
Age			-0.00132			-0.000164			-0.000645
			(0.00336)			(0.00197)			(0.00156)
State of Residence			0.00777**			0.00512**			0.00403**
			(0.00359)			(0.00206)			(0.00161)
Restrictions in the State			-0.156			-0.102			-0.0790
			(0.111)			(0.0658)			(0.0507)
Constant							3.938***	2.336***	2.420***
							(0.0332)	(0.190)	(0.234)
Observations	2074	1837	1828	2074	1837	1828	2074	1837	1828
Adjusted R^2							0.001	0.148	0.197

Standard errors in parentheses

* $p < 0.10$, ** $p < 0.05$, *** $p < 0.01$

Table A6: Determinants of support for keeping shops closed. Coefficients estimated through ordered Logit regressions (Columns 1-3), ordered Probit regressions (Columns 4-6) and Ordinary Least Squares (OLS) regressions (Columns 7-9). Standard errors are reported in parentheses. All coefficients for the control variables are reported.

	(1) Support for Closing Businesses	(2) Support for Closing Businesses	(3) Support for Closing Businesses	(4) Support for Closing Businesses	(5) Support for Closing Businesses	(6) Support for Closing Businesses	(7) Support for Closing Businesses	(8) Support for Closing Businesses	(9) Support for Closing Businesses
In Linear Group	0.0406	-0.378**	-0.424**	0.0251	-0.181*	-0.213**	0.0261	-0.125	-0.121
	(0.0822)	(0.161)	(0.168)	(0.0491)	(0.0934)	(0.0958)	(0.0512)	(0.0880)	(0.0842)
Worry About Health Crisis		0.997***	1.067***		0.550***	0.592***		0.527***	0.543***
		(0.0524)	(0.0567)		(0.0285)	(0.0313)		(0.0291)	(0.0282)
COVID-19 News Checking		0.0288	0.0748		0.0145	0.0387		-0.00802	0.0147
		(0.0461)	(0.0477)		(0.0268)	(0.0277)		(0.0228)	(0.0219)
Male		-0.112	-0.0890		-0.0630	-0.0532		-0.0252	-0.0200
		(0.0956)	(0.0984)		(0.0561)	(0.0574)		(0.0468)	(0.0452)
Understanding Q.1: Real Data		0.131	0.132		0.0560	0.0543		0.0429	0.0319
		(0.109)	(0.111)		(0.0637)	(0.0646)		(0.0550)	(0.0533)
Confidence in Understanding Q.1		0.00955***	0.00842**		0.00457**	0.00406*		0.00393**	0.00337*
		(0.00367)	(0.00371)		(0.00213)	(0.00215)		(0.00198)	(0.00186)
Understanding Q.2: Hypothetical		0.300*	0.348**		0.138	0.168*		0.0909	0.0949
		(0.168)	(0.175)		(0.0974)	(0.0996)		(0.0923)	(0.0882)
Confidence in Understanding Q.2		-0.000421	-0.000228		0.000629	0.000817		-0.000418	-0.000429
		(0.00375)	(0.00379)		(0.00217)	(0.00220)		(0.00202)	(0.00190)
Accurate Prediction		0.480**	0.450**		0.293***	0.262**		0.210**	0.172*
		(0.190)	(0.193)		(0.113)	(0.115)		(0.0979)	(0.0903)
Unreasonable Prediction		0.0871	0.0806		0.0701	0.0529		0.0335	0.0168
		(0.183)	(0.186)		(0.110)	(0.111)		(0.0954)	(0.0884)
Confidence in Prediction		-0.00451*	-0.00426*		-0.00266**	-0.00268*		-0.00251**	-0.00236**
		(0.00234)	(0.00237)		(0.00135)	(0.00138)		(0.00122)	(0.00115)
Democrat			0.545***			0.310***			0.190***
			(0.116)			(0.0673)			(0.0513)
Republican			-0.491***			-0.298***			-0.299***
			(0.120)			(0.0701)			(0.0651)
Worry About Economic Crisis			-0.494***			-0.289***			-0.257***
			(0.0613)			(0.0350)			(0.0265)
Live in city with <50K People			0.0314			0.0310			0.0447
			(0.107)			(0.0625)			(0.0491)
Live in city with >500K People			0.0230			-0.00403			-0.00391
			(0.129)			(0.0748)			(0.0622)
Education			-0.0258			-0.0121			-0.0107
			(0.0379)			(0.0220)			(0.0178)
Age			-0.00105			-0.00115			-0.00126
			(0.00356)			(0.00204)			(0.00172)
State of Residence			0.00274			0.00158			0.000767
			(0.00367)			(0.00217)			(0.00152)
Restrictions in the State			-0.0175			-0.00715			-0.000582
			(0.117)			(0.0688)			(0.0521)
Constant							4.067***	1.804***	2.901***
							(0.0356)	(0.183)	(0.240)
Observations	2074	1837	1828	2074	1837	1828	2074	1837	1828
Adjusted R^2							-0.000	0.233	0.304

Standard errors in parentheses
* $p < 0.10$, ** $p < 0.05$, *** $p < 0.01$

Table A7: Determinants for suggested reopening day. Columns 1-3 report coefficients obtained through Ordinary Least Squares (OLS) regressions, Columns 3-6 report coefficients obtained through Logit regressions (on the months before reopening) and Columns 7-9 report coefficients from OLS regressions (on the months before reopening). Standard errors are reported in parentheses. All coefficients for the control variables are reported.

	(1) Days Until Reopening Businesses	(2) Days Until Reopening Businesses	(3) Days Until Reopening Businesses	(4) Months until reopening	(5) Months until reopening	(6) Months until reopening	(7) Months until reopening	(8) Months until reopening	(9) Months until reopening
In Linear Group	2.295	17.38**	14.65**	0.150*	0.396**	0.335*	3.044	18.60***	16.00**
	(3.133)	(7.069)	(7.010)	(0.0790)	(0.174)	(0.175)	(3.054)	(6.843)	(6.804)
Worry About Health Crisis		12.45***	13.14***		0.682***	0.701***		11.43***	11.94***
		(1.789)	(1.910)		(0.0523)	(0.0548)		(1.729)	(1.858)
COVID-19 News Checking		3.071*	3.932**		0.0710*	0.120***		3.296**	4.147**
		(1.609)	(1.664)		(0.0420)	(0.0430)		(1.567)	(1.624)
Male		10.53***	9.169***		0.351***	0.340***		11.20***	10.07***
		(3.377)	(3.335)		(0.0893)	(0.0889)		(3.305)	(3.267)
Understanding Q.1: Real Data		-1.236	-0.517		-0.0111	0.0113		-1.394	-0.663
		(4.088)	(4.112)		(0.104)	(0.105)		(3.996)	(4.027)
Confidence in Understanding Q.1		0.109	0.0996		0.00595*	0.00528		0.0937	0.0862
		(0.128)	(0.129)		(0.00352)	(0.00360)		(0.122)	(0.123)
Understanding Q.2: Hypothetical		-18.05**	-15.87**		-0.353*	-0.309*		-18.06***	-16.04**
		(7.177)	(7.125)		(0.181)	(0.183)		(6.976)	(6.941)
Confidence in Understanding Q.2		-0.310**	-0.299**		-0.00709*	-0.00716*		-0.296**	-0.285**
		(0.138)	(0.139)		(0.00363)	(0.00375)		(0.130)	(0.132)
Accurate Prediction		10.58*	9.343		0.368**	0.337**		10.95*	9.881
		(6.297)	(6.295)		(0.165)	(0.161)		(6.155)	(6.164)
Unreasonable Prediction		6.590	4.787		0.0971	0.0854		6.706	5.081
		(6.060)	(6.071)		(0.160)	(0.157)		(5.930)	(5.953)
Confidence in Prediction		0.216***	0.205**		0.0000901	-0.000193		0.211***	0.198**
		(0.0799)	(0.0811)		(0.00205)	(0.00207)		(0.0782)	(0.0794)
In Linear Group			0						
			(.)						
Democrat			0.107			0.186*			1.055
			(3.683)			(0.102)			(3.592)
Republican			1.912			-0.316**			3.064
			(4.675)			(0.125)			(4.573)
Worry About Economic Crisis			-3.597*			-0.233***			-2.972
			(1.981)			(0.0520)			(1.923)
Live in city with <50K People			6.259*			0.140			5.503
			(3.626)			(0.0976)			(3.565)
Live in city with >500K People			9.164**			0.184			7.363*
			(4.394)			(0.113)			(4.271)
Education			-1.798			-0.0753**			-1.894
			(1.319)			(0.0341)			(1.282)
Age			-0.151			-0.00831**			-0.153
			(0.116)			(0.00327)			(0.113)
State of Residence			-0.00686			0.000271			0.00475
			(0.127)			(0.00298)			(0.127)
Restrictions in the State			-1.382			-0.00498			-0.420
			(4.178)			(0.110)			(4.126)
In Linear Group						0			
						(.)			
In Linear Group									0
									(.)
Constant	65.38***	-0.312	24.09				54.48***	-7.962	12.68
	(2.156)	(11.67)	(16.94)				(2.105)	(11.39)	(16.53)
Observations	2061	1828	1819	2074	1837	1828	2074	1837	1828
Adjusted R^2	-0.000	0.055	0.056				-0.000	0.053	0.053

Standard errors in parentheses
* $p < 0.10$, ** $p < 0.05$, *** $p < 0.01$

Table A8: Determinants of support for a tax to finance masks' distribution. The coefficients are estimated through ordered Logit regressions (Columns 1-3), ordered Probit regressions (Columns 4-6) and ordinary least squares (OLS) regressions (Columns 7-9). Standard errors are reported in parentheses. All coefficients for the control variables are reported.

	(1)<br>Support for<br>Mask-Buying Tax	(2)<br>Support for<br>Mask-Buying Tax	(3)<br>Support for<br>Mask-Buying Tax	(4)<br>Support for<br>Mask-Buying Tax	(5)<br>Support for<br>Mask-Buying Tax	(6)<br>Support for<br>Mask-Buying Tax	(7)<br>Support for<br>Mask-Buying Tax	(8)<br>Support for<br>Mask-Buying Tax	(9)<br>Support for<br>Mask-Buying Tax
In Linear Group	-0.0218	0.307**	0.305**	-0.00837	0.163*	0.156*	-0.0130	0.175*	0.162
	(0.0781)	(0.151)	(0.153)	(0.0465)	(0.0882)	(0.0892)	(0.0608)	(0.103)	(0.103)
Worry About Health Crisis		0.481***	0.471***		0.280***	0.276***		0.307***	0.295***
		(0.0506)	(0.0538)		(0.0294)	(0.0313)		(0.0335)	(0.0346)
Likelihood to Wear Masks		0.648***	0.617***		0.376***	0.362***		0.410***	0.386***
		(0.0496)	(0.0504)		(0.0288)	(0.0294)		(0.0304)	(0.0305)
COVID-19 News Checking		0.0403	0.0682		0.0213	0.0384		0.0184	0.0393
		(0.0423)	(0.0434)		(0.0249)	(0.0255)		(0.0278)	(0.0282)
Male		0.0372	0.0455		0.0198	0.0280		0.0208	0.0284
		(0.0883)	(0.0896)		(0.0523)	(0.0530)		(0.0582)	(0.0576)
Understanding Q.1: Real Data		0.152	0.169*		0.0865	0.0912		0.0818	0.0886
		(0.101)	(0.102)		(0.0597)	(0.0601)		(0.0672)	(0.0665)
Confidence in Understanding Q.1		0.00648*	0.00602*		0.00343*	0.00287		0.00241	0.00181
		(0.00352)	(0.00353)		(0.00205)	(0.00205)		(0.00241)	(0.00239)
Understanding Q.2: Hypothetical		-0.454***	-0.452***		-0.247***	-0.239**		-0.273**	-0.258**
		(0.157)	(0.159)		(0.0917)	(0.0927)		(0.108)	(0.107)
Confidence in Understanding Q.2		-0.0108***	-0.0112***		-0.00577***	-0.00583***		-0.00543**	-0.00546**
		(0.00362)	(0.00364)		(0.00209)	(0.00210)		(0.00253)	(0.00252)
Accurate Prediction		0.186	0.141		0.0999	0.0782		0.129	0.103
		(0.184)	(0.185)		(0.107)	(0.108)		(0.127)	(0.125)
Unreasonable Prediction		0.165	0.147		0.0870	0.0792		0.114	0.106
		(0.179)	(0.180)		(0.104)	(0.105)		(0.123)	(0.122)
Confidence in Prediction		0.00675***	0.00734***		0.00345***	0.00367***		0.00423***	0.00435***
		(0.00217)	(0.00220)		(0.00126)	(0.00127)		(0.00148)	(0.00146)
Democrat			0.378***			0.216***			0.250***
			(0.104)			(0.0612)			(0.0694)
Republican			-0.261**			-0.151**			-0.188**
			(0.116)			(0.0683)			(0.0755)
Worry About Economic Crisis			-0.0979*			-0.0676**			-0.0791**
			(0.0538)			(0.0315)			(0.0349)
Live in city with <50K People			0.115			0.0759			0.0974
			(0.0983)			(0.0580)			(0.0640)
Live in city with >500K People			0.0488			0.0397			0.0531
			(0.119)			(0.0697)			(0.0773)
Education			-0.0209			-0.0176			-0.0238
			(0.0345)			(0.0204)			(0.0220)
Age			-0.00942***			-0.00582***			-0.00748***
			(0.00325)			(0.00191)			(0.00214)
State of Residence			-0.00313			-0.00186			-0.00270
			(0.00341)			(0.00197)			(0.00227)
Restrictions in the State			-0.122			-0.0561			-0.0612
			(0.108)			(0.0640)			(0.0701)
Constant							3.251***	0.176	1.101***
							(0.0421)	(0.214)	(0.310)
Observations	2072	1834	1825	2072	1834	1825	2072	1834	1825
Adjusted R^2							-0.000	0.230	0.258

Standard errors in parentheses
* $p < 0.10$, ** $p < 0.05$, *** $p < 0.01$

Table A9: Determinants of likelihood to wear a mask when going out if provided with one. The coefficients are estimated through ordered Logit regressions (Columns 1-3), ordered Probit regressions (Columns 4-6) and Ordinary Least Squares (OLS) regressions (Columns 7-9). Standard errors are reported in parentheses. All coefficients for the control variables are reported

	(1) Likelihood Wear Masks	(2) Likelihood Wear Masks	(3) Likelihood Wear Masks	(4) Likelihood Wear Masks	(5) Likelihood Wear Masks	(6) Likelihood Wear Masks	(7) Likelihood Wear Masks	(8) Likelihood Wear Masks	(9) Likelihood Wear Masks
In Linear Group	0.00311	-0.314**	-0.350**	0.00492	-0.195**	-0.209**	0.00611	-0.150**	-0.161**
	(0.0818)	(0.157)	(0.160)	(0.0488)	(0.0929)	(0.0942)	(0.0482)	(0.0694)	(0.0703)
Worry About Health Crisis		0.907***	0.908***		0.512***	0.511***		0.467***	0.463***
		(0.0514)	(0.0547)		(0.0281)	(0.0301)		(0.0284)	(0.0292)
COVID-19 News Checking		0.138***	0.129***		0.0840***	0.0794***		0.0450**	0.0422**
		(0.0458)	(0.0472)		(0.0266)	(0.0274)		(0.0212)	(0.0212)
Male		-0.255***	-0.270***		-0.151***	-0.163***		-0.0940**	-0.105**
		(0.0944)	(0.0963)		(0.0555)	(0.0564)		(0.0435)	(0.0434)
Understanding Q.1: Real Data		0.0281	0.0136		0.0238	0.0116		0.0126	0.00199
		(0.109)	(0.110)		(0.0637)	(0.0643)		(0.0512)	(0.0509)
Confidence in Understanding Q.1		0.00571	0.00493		0.00338	0.00305		0.00309	0.00293
		(0.00372)	(0.00378)		(0.00211)	(0.00213)		(0.00198)	(0.00194)
Understanding Q.2: Hypothetical		0.189	0.237		0.111	0.132		0.0930	0.111
		(0.164)	(0.167)		(0.0971)	(0.0984)		(0.0741)	(0.0747)
Confidence in Understanding Q.2		0.00250	0.00272		0.00164	0.00161		0.000816	0.000547
		(0.00380)	(0.00384)		(0.00216)	(0.00217)		(0.00200)	(0.00196)
Accurate Prediction		0.435**	0.431**		0.249**	0.236**		0.179*	0.163*
		(0.187)	(0.188)		(0.110)	(0.110)		(0.0975)	(0.0957)
Unreasonable Prediction		0.497***	0.493***		0.279***	0.268**		0.181*	0.165*
		(0.183)	(0.184)		(0.107)	(0.108)		(0.0952)	(0.0938)
Confidence in Prediction		0.00211	0.00276		0.00147	0.00173		0.000839	0.00103
		(0.00227)	(0.00230)		(0.00133)	(0.00135)		(0.00111)	(0.00109)
Democrat			0.161			0.104			0.0644
			(0.113)			(0.0659)			(0.0503)
Republican			-0.384***			-0.208***			-0.175***
			(0.121)			(0.0704)			(0.0616)
Worry About Economic Crisis			-0.132**			-0.0799**			-0.0898***
			(0.0573)			(0.0330)			(0.0277)
Live in city with <50K People			0.0832			0.0430			0.0391
			(0.104)			(0.0610)			(0.0497)
Live in city with >500K People			0.588***			0.339***			0.242***
			(0.129)			(0.0750)			(0.0559)
Education			-0.0767**			-0.0355			-0.0144
			(0.0374)			(0.0216)			(0.0178)
Age			0.00713**			0.00425**			0.00282*
			(0.00350)			(0.00204)			(0.00153)
State of Residence			0.0170***			0.0102***			0.00640***
			(0.00395)			(0.00225)			(0.00136)
Restrictions in the State			-0.154			-0.0974			-0.0853
			(0.114)			(0.0672)			(0.0565)
Constant							4.090***	1.651***	2.130***
							(0.0335)	(0.189)	(0.254)
Observations	2072	1835	1826	2072	1835	1826	2072	1835	1826
Adjusted R^2							-0.000	0.227	0.255

Standard errors in parentheses
* $p < 0.10$, ** $p < 0.05$, *** $p < 0.01$

2. Questionnaire

Table A10: Survey Questions - Part I

**Questionnaire page**	**Worry, Policy and Attitudinal Questions**	**Figure Shown**
1	Respondents are shown a graph that shows data on deaths from COVID-19 on either a linear scale or a logarithmic scale. Below the figure we provide information on how to read the scale and on the total number of deaths as of April 18th 2020	Figure 1 - Group A linear scale and Group B logarithmic scale
2	How worried are you about the health and economic crises caused by the coronavirus pandemic?	None
2a	How worried are you about the HEALTH crisis in the US? Rate your level of worry from 1 (Not worried at all) to 5 (Extremely worried)	None
2b	How worried are you about the ECONOMIC crisis in the US? Rate your level of worry from 1 (Not worried at all) to 5 (Extremely worried)	None
3	Do you agree that all NON-ESSENTIAL businesses should be closed? Essential businesses are supermarkets, pharmacies, etc. (Strongly Disagree - Strongly Agree)	None
4	Until when do you think non-essential businesses should closed? Please insert a date below. (Month, Day)	None
5a	It has been suggested that governments should send protective masks to their citizens. If the government sent you a supply of masks, how often would you wear them when you go outside? (Never - Always)	None
5b	How strongly would you support a tax that finances the distribution of masks for everyone in your state? (Strongly oppose - Strongly support)	None

Table A11: Survey Questions

Questionnaire page	Understanding Questions	Figure Shown
6a	In this question we encourage you to give your best guess. Approximately, how many TOTAL DEATHS do you think there will be by on April 25th 2020? Please insert a NUMBER below:	Figure 1 - Group A linear scale and Group B logarithmic scale
6b	How confident do you feel in your answer? (1-100%)	Figure 1 - Group A linear scale and Group B logarithmic scale
7a	Looking at this figure, did the total number of deaths increase MORE between March 31st and April 6th or between April 6th and April 12th? (It increased more between March 31st and April 6th; It increased more between 6th and April 12th; The number of new cases was the same in the two weeks, I don't know)	Figure 1 - Group A linear scale and Group B logarithmic scale
7b	How confident do you feel in your answer? (1-100	Figure 1 - Group A linear scale and Group B logarithmic scale
8a	When was there a LARGER DIFFERENCE between the number of men and women dying after suffering infection Type Z? (From week 2 to week 3, From week 5 to week 6, From week 9 to week 10, From week 13 to week 14, I do not know)	Figure from Okan (2016)
8b	How confident do you feel in your answer? (1-100	Figure from Okan (2016)

Table A12: Survey Questions - Part III

Questionnaire page	Demographics	Figure Shown
9	How often do you read the news about the coronavirus pandemic? Please give us your best guess. (Less than once a day - Five or more times a day)	None
10	In which state do you currently reside? (Choice from menu)	None
11	How many people live in your town/city? (Less than 50,000; Between 50,000 and 500,000; More than 500,000; I don't know)	None
12	What is your year of birth? (Free answer)	None
13	What is the highest level of school you have completed or the highest degree you have received? (Choice from type of degree list)	None
14	What is your gender? (Male, Female, Other, Prefer not to declare)	None
15	Information about your income is very important for us to analyze data. Would you please give your best guess? Please indicate the answer that includes your entire household income in 2019 before taxes. (Choice between income brackets)	None
16	Generally speaking, do you usually think of yourself as a Republican, a Democrat, an Independent, or something else? (Republican, Democrat, Independent, Other, No preference)	None
17	Which device did you use to complete this survey? (I used a laptop or a desktop computer, I used a smartphone, I used a tablet, Other)	None
