# Supplementary material for: The scale of COVID‐19 graphs affects understanding, attitudes, and policy preferences
Source: Health Econ. 2020 Aug 25;29(11):1482–94. doi: 10.1002/hec.4143 (PMC7461444; doi:10.1002/hec.4143)
Supplement: Supplementary file 2 — Supplementary Material [file HEC-29-1482-s001.pdf]

~~COVID-19 Data: The Logarithmic Scale Misinforms the Public and Affects Policy Preferences~~The Scale of COVID-19 Graphs Affects Understanding, Attitudes, and Policy Preferences

Abstract

Mass media routinely present data on COVID-19 diffusion with graphs that use either a log scale or a linear scale. We show that the choice of the scale adopted on these graphs has important consequences on how people understand and react to the information conveyed. In particular, we find that when we show the number of COVID-19 related deaths on a logarithmic scale, people have a less accurate understanding of how the pandemic has developed, make less accurate predictions on its evolution, and have different policy preferences than when they are exposed to a linear scale. Consequently, merely changing the scale the data is presented on can alter public policy preferences and the level of worry about the pandemic, despite the fact that people are routinely exposed to ~~a lot of~~ COVID-19 related information. ~~Reducing misinformation~~ Providing the public with information in ways they understand better can help improving the response to COVID-19, thus, mass media and policymakers communicating to the general public should always describe the evolution of the pandemic using a graph on a linear scale, at least as a default option~~or at least they should show both scales~~. Our results suggest that framing matters when communicating to the public~~More generally, our results confirm that policymakers should not only care about *what* information to communicate, but also about *how* to do it, as even small differences in the framing of data can have a significant impact.~~

Keywords: COVID-19, Public Understanding, Framing, Media

1. Introduction

The coronavirus disease 2019 (COVID-19) pandemic is a formidable challenge. Absent a cure or a vaccine, it is crucial that people are adequately informed about the pandemic (Everett et al., 2020), so that they stand behind policies that aim to minimize the spread of the virus and adopt behaviors that can limit the risk of contagion (Bursztyn et al.,

2020). However, research has shown the challenges of communicating scientific facts in a way that effectively conveys essential information to the general public (Pidgeon and Fischhoff, 2011). In this article, we highlight the importance of this problem by focusing on one of the most basic pieces of information relative to the pandemic: the number of deaths.

To provide information on the diffusion of the virus, mass media routinely publish graphs that depict the evolution in the number of COVID-19 related deaths in a given area. Many of these graphs present quantities on the Y-axis on either a linear scale (TheWashingtonPost, 2020, Vox, 2020) or a logarithmic scale (Guardian, 2020, Financial-Times, 2020, NewYorkTimes, 2020a). The New York Times, for instance, has explained that the logarithmic scale helps better visualize exponential growth (NewYorkTimes, 2020b).This follows advice given by epidemiology journals (Gladen, 1983, Levine et al., 2010) and data visualization handbooks (Kosslyn, 2006). However, what might be true for conveying information among experts might not hold when issuing information to a broader audience. The principle that logarithmic scales are better suited for exponential growth does not hold true if readers do not, in fact, comprehend them.

We show that scale choice has important consequences on how people understand and react to the information conveyed. In particular, we find that when people are exposed to a logarithmic scale they have a less accurate understanding of how the pandemic unfolded until now, make less accurate predictions on its future, and have different attitudes and policy preferences than when they are exposed to a linear scale. Another study (Ryan and Evers, 2020) carried out a week after ours, confirms our finding that the scale of the graph affects policy preferences and that people have problems understanding logarithms. Instead, a study with Canadian respondents finds that the scale of the graph has no impact on respondents (Sevi et al., 2020).[1]. Previous studies have already shown that even experts have problems understanding graphs that use the logarithmic scale (Menge et al., 2018, Heckler et al., 2013). However, unlike most stud-

[1]However, their study uses a "catch all" question for pessimism and one on policy preferences. These catch all questions might be unable to capture the nuanced impact of graph scale on policies and attitudes that we observe. For instance, we observe an impact on worry for the health crisis, but *not* on worry for the economic crisis.

ies on graph comprehension we test understanding of graphs that represents real world highly salient data about which the public is likely to have ample background information and to care deeply. The obvious relevance of the data depicted in the graphs also allows us to test the impact of the scale in which the data is plotted on preferences about important policy issues. ~~This result is consistent with existing evidence that even engineering students and specialized scientists have trouble understanding information conveyed in logarithmic scale graphs (Menge et al., 2018, Menge et al., 2013) Since reducing misinformation~~ Since providing the public with clear information can help improving the response to COVID-19 (Van Bavel et al., 2020), mass media and policymakers should present data on the evolution of the pandemic using a graph on a linear scale, at least as a default option~~or at least they should show both scales~~.

2. Experiment

We devised a double-blind experiment approved by the Yale IRB to test people's graph comprehension and its effects on attitudes and policy preferences.~~The experiment was approved by Yale's Institutional Review Board, and we asked all participants to confirm that they were 18 years old or older at the time of taking the survey and giving their informed consent before participating. Those clicking no on any of the two statements were not allowed to answer any question. All participants were recruited through Cloud Research, while the survey was structured on and administered via Qualtrics, where we were able to download the anonymized data.~~ We recruited a sample of approximately $n = 2000$ (after exclusion criteria, with no regression with less than 1825 observations) U.S. residents on Cloud Research. Half of them were randomly assigned to the Linear Group, in which they were shown the evolution of COVID-19 deaths in the U.S. on a linear scale. The other half were assigned to the Log Group, in which participants saw the same data, but plotted on a logarithmic scale. The graphs were taken from the popular website www.worldometers.info (See Fig 1). We asked respondents three sets of questions: (*i*) attitudes and policy preferences, (*ii*) graph understanding, and (*iii*) standard demographic questions. In the supplementary material, we report the questions we asked and the order in which they were asked.

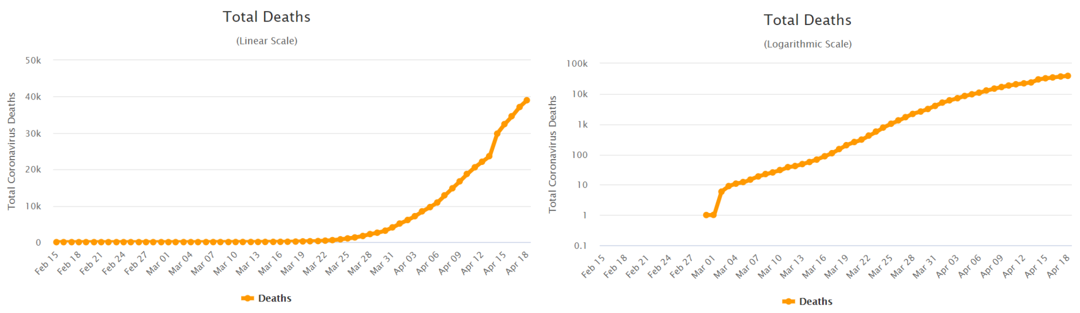

Figure 1: COVID-19 Related Deaths in United States Between February 15th and April 18th in a linear scale (left panel) and in a log scale (right panel). Source: www.worldometers.info

The analyses can be grouped into: 1) determinants of worry, 2) policy preferences and 3) differences in understanding. In all three cases our primary variable of interest is ”linear”, a binary taking value 1 whenever the participant was exposed to the linear scale graphs, and 0 otherwise.

We start by showing participants in the two groups the graph plotting the evolution of the total number of deaths on the scale to which they were randomly assigned. Then we ask respondents in the two groups to indicate how worried they are about the health crisis and the economic crisis caused by COVID-19 on a five points Likert scale from “not worried at all” to “extremely worried”. Second, we ask respondents about their preferences on some policies that many States have adopted to mitigate the spread of COVID-19. In the first pair of policy questions we ask whether they support the policy of closing non-essential businesses (five points Likert scale from “strongly disagree” to “strongly agree”), and until which date they would keep these businesses closed. In the second pair of policy questions we ask participants how often they would use a mask if the government sent a supply (five points Likert scale from “never” to “always”). Moreover,, we ask whether they would support a tax that finances the distribution of masks for everyone in their State (five points Likert scale from “strongly oppose” to “strongly support”).

We then turn to test respondents’ understanding of the graphs. To increase external validity and to avoid priming respondents, we ask attitudes and policy preferences *before* testing understanding. This allows us to obtain respondents’ policy preferences before they are asked to think thoroughly about the graph and its meaning in a way that they

would be unlikely to do when reading actual news.

We test understanding of graphs by asking three questions. First, we show them the COVID-19 graph on the scale that they had been assigned and ask them whether the number of deaths increased more between March 31st and April 6th or between April 6th and April 12th. Second, we show them a graph describing non-COVID-19 related data on the number of deaths from an hypothetical infection Z (taken from Okan et al. (2016)) and asked them a similar question. As for the first graph shown to participants, people in the Linear Group saw the data plotted on a linear scale, whereas respondents in the Log Group saw data plotted on a logarithmic one. The goal of this question was to test whether respondents' ability to answer correctly the first question depended on prior information on COVID-19, or on a correct understanding of the scale on which their graphs are plotted.

Third, we test whether respondents can make predictions based on the curve. In particular, we ask them to make a prediction on the total number of deaths on April 25th, one week after we launched the experiment.

Predicting the number of COVID-19 related deaths in a week is very difficult, but some predictions are more reasonable than others. We forecast the number of total deaths on April 25th using an ARIMA model, a standard forecasting method that has already been used to predict COVID-19 diffusion (Benvenuto et al., 2020). We use a ARIMA (0,2,1), as simulations show that it offers the best fit for the data, and forecast the number of cases and its 95% and 99% confidence intervals (CIs). On the 18th of April the number of deaths was $39,014$. The 95% CI forecasted using the ARIMA(0,2,1) ranges from $49,203.15$ to $62,559.27$, whereas the 99% CI ranges from $46,895.47$ to $64,685.95$. We remark that the actual number of deaths on the 25th of April was $54,256$, while our ARIMA predicted $55,791$ deaths predicted model. This is well within the CIs we consider.

We use these CIs to divide predictions in three groups. In the first group, we include the predictions that fall within the forecast 95% confidence interval ("accurate range"). We consider these predictions "accurate". In the second group, we include the predictions that fall within the 99% confidence interval, but outside the 95% confidence

interval ("unlikely range"). We refer to these predictions as "unlikely". Last, we consider the predictions that fall outside the 99% confidence interval ("unreasonable range") as "unreasonable".

Additionally, for each of the understanding questions we asked how confident respondents were about their answers. The level of confidence is important as it can shed some light on how much weight people will attach to the information represented in the graph.

We concluded by collecting standard demographic information on the respondents.

3. Results and Discussion

Table 1 describes the characteristics of our sample. Figures 2-3 and Tables 2-3 show that people in the Linear Group understand the graphs better and make better predictions. The Log Group gives predictions that are higher and are on average unreasonable. Therefore, using linear scale graphs reduces the risk of confusing the public.

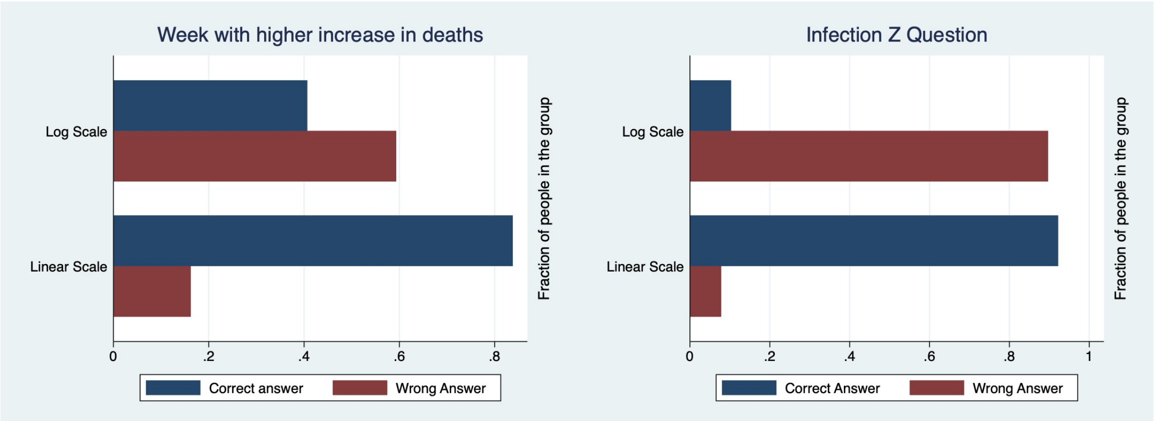

Figure 2: The left panel reports the percentage of correct and incorrect answers provided by the members of the two groups to the understanding question related to COVID-19 real world data. The right panel reports the percentage of correct and incorrect answers provided by the members of the two groups to the understanding question related to Infection Z hypothetical data

Moreover, the scale also impacts people level of worry for the health crisis (but not for the economic crisis) and their policy preferences. People in the Linear Group are more worried about the health crisis (see Table 4), and prefer that non-essential businesses remain closed for longer (Table 5). However, they support less strongly the idea of closing non-essential business in the first place (Table 5), and would wear government-supplied masks less often (Table 6). These results are statistically significant and robust

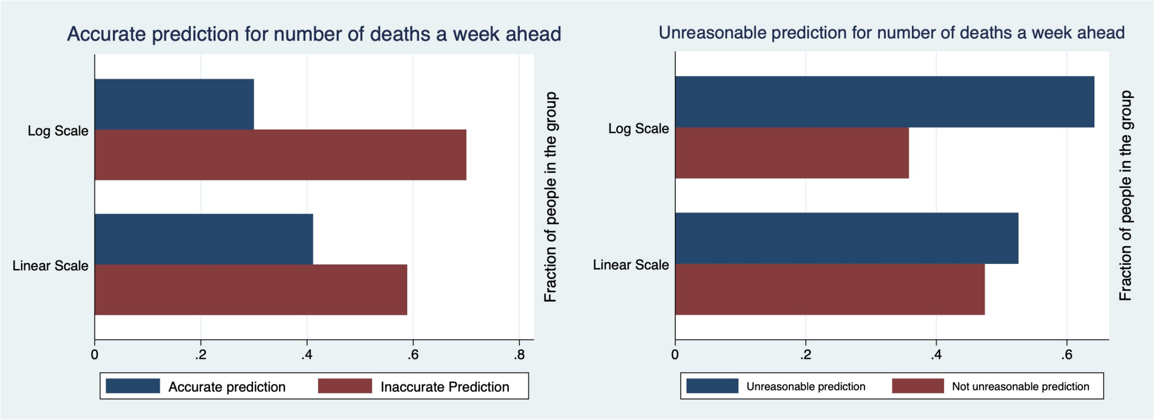

Figure 3: The left panel reports the percentage of accurate and inaccurate (i.e. not accurate) predictions provided by the members of the two groups. The right panel reports the unreasonable and reasonable (i.e. not unreasonable) predictions provided by the members of the two groups.

to a series of different controls and specifications (the regressions presented use Logit and OLS and the results are robust to different sets of controls). The odds ratios show that the magnitude of the effects is non-negligible (Table 7).

These findings are remarkable because the data underlying the graphs is identical. Merely changing the scale can alter public policy preferences and the level of worry, despite the endless flow of COVID-19 related information to which everyone is exposed.

We cannot know the mechanism leading to these preferences, but we advance the conjecture that the shape of the curves could explain these findings. The flat logarithmic curve can give the impression that we reached a plateau and that, while the present situation is very serious, things are about to get better soon. Thus respondents in the Log Group might be less worried because they feel that the end of the pandemic is near. For the same reason, they could strongly support closing non essential businesses now, i.e. during the peak, but could want to reopen them as soon as the peak is over. Moreover, they might concentrate the use of masks during the peak. As the Log Group thinks we are at the peak, they could also expect a very high number of deaths in the short term, which would also explain their strong support to wear masks and to keep business closed.

Vice versa, the linear curve is constantly growing with no sign of improvement, hence it might give the impression that the crisis will go on for long and will be very serious. Consequently, people in the Linear Group might be more worried and wish to reopen non-essential businesses later. However, they could support closing non-essential businesses

relatively less, because they believe that the pandemic will last for a long time, and non-essential businesses cannot remain closed for too long. However, if the decision taken is to close non-essential businesses, they might feel that it would be pointless to do it for a short period of time. They would apply a similar logic to masks. As they believe that the pandemic will last for a long time, they could use them less frequently to ration them.

Regardless of the reasons behind our findings, it is noteworthy that changing the scale can alter policy preferences, intentions to adopt precautionary measures, and level of worry for the health consequences of the pandemic. Given that the scale affects policy preferences and that people have significant problems understanding the logarithmic scale, our findings suggests that representing data on a linear scale is preferable. Garfin et al. (2020) noted that during a public health crisis, the general public relies on the media to convey accurate and understandable information, so that it can take informed decisions regarding health protective behaviors. Absent information of this kind, people cannot form informed preferences or take informed decisions. Moreover, unclear information conveyed by the media could undermine how much people trust science, which is a key predictor of compliance with COVID-19 guidelines (Brzezinski et al., 2020, Phlol and Musil, 2020).

4. Tables

Table 1: Frequency Table for Demographic variables: Number, Percentage and Cumulative Percentage of respondents for the following variables: Age, Education, Income, Political orientation, Gender, Live in city with less than 50K people, Live in city with more than 500K people. Column 1 shows the overall distribution, Column 2 shows the distribution for the Linear Group and Column 3 the one for the Log Group.

	**Graph shown**								
	**Log Scale**			**Linear Scale**			**Total**		
	No.	Col %	Cum %	No.	Col %	Cum %	No.	Col %	Cum %
**Age**									
18-25 years old	126	11.6	11.6	122	12.4	12.4	248	12.0	12.0
26-35 years old	351	32.3	43.9	309	31.3	43.7	660	31.8	43.8
36-45 years old	234	21.5	65.4	237	24.0	67.7	471	22.7	66.5
46-55 years old	182	16.7	82.2	150	15.2	82.9	332	16.0	82.5
56-65 years old	129	11.9	94.0	107	10.8	93.7	236	11.4	93.9
66-75 years old	57	5.2	99.3	52	5.3	99.0	109	5.3	99.1
>75 years old	8	0.7	100.0	10	1.0	100.0	18	0.9	100.0
**Education**									
Less than high school degree	4	0.4	0.4	5	0.5	0.5	9	0.4	0.4
High school graduate (diploma or equivalent)	88	8.1	8.5	83	8.4	8.9	171	8.3	8.7
Some college but no degree	210	19.3	27.8	168	17.0	26.0	378	18.2	26.9
Associate degree in college (2-year)	97	8.9	36.7	101	10.2	36.2	198	9.6	36.5
Bachelor's degree in college	478	44.0	80.8	402	40.8	77.0	880	42.5	79.0
Master's degree or Professional Degree (JD, MD, etc)	190	17.5	98.3	203	20.6	97.6	393	19.0	97.9
Doctoral degree	19	1.7	100.0	24	2.4	100.0	43	2.1	100.0
**Income**									
Less than $10,000	48	4.4	4.4	36	3.7	3.7	84	4.1	4.1
$10,000 to $19,999	64	5.9	10.3	56	5.7	9.3	120	5.8	9.9
$20,000 to $29,999	75	6.9	17.2	96	9.8	19.1	171	8.3	18.1
$30,000 to $39,999	120	11.1	28.3	88	8.9	28.0	208	10.1	28.2
$40,000 to $49,999	108	10.0	38.2	104	10.6	38.6	212	10.2	38.4
$50,000 to $59,999	111	10.2	48.5	103	10.5	49.1	214	10.3	48.8
$60,000 to $69,999	100	9.2	57.7	85	8.6	57.7	185	8.9	57.7
$70,000 to $79,999	100	9.2	66.9	75	7.6	65.3	175	8.5	66.2
$80,000 to $89,999	58	5.3	72.3	68	6.9	72.3	126	6.1	72.3
$80,000 to $89,999	60	5.5	77.8	71	7.2	79.5	131	6.3	78.6
$90,000 to $99,999	164	15.1	92.9	128	13.0	92.5	292	14.1	92.7
$150,000 or more	77	7.1	100.0	74	7.5	100.0	151	7.3	100.0
**Political orientation**									
Other	352	32.4	32.4	292	29.6	29.6	644	31.1	31.1
Democrat	441	40.6	73.0	426	43.2	72.7	867	41.8	72.9
Republican	294	27.0	100.0	269	27.3	100.0	563	27.1	100.0
**Total**	1087	100.0		987	100.0		2074	100.0	
**Gender**									
Other/Prefer not to declare	8	0.7	0.7	14	1.4	1.4	22	1.1	1.1
Female	571	52.5	53.3	524	53.1	54.5	1095	52.8	53.9
Male	508	46.7	100.0	449	45.5	100.0	957	46.1	100.0
**Live in city with <50K People**									
No	680	62.6	62.6	601	60.9	60.9	1281	61.8	61.8
Yes	407	37.4	100.0	386	39.1	100.0	793	38.2	100.0
**Total**	1087	100.0		987	100.0		2074	100.0	
**Live in city with >500K People**									
No	851	78.3	78.3	769	77.9	77.9	1620	78.1	78.1
Yes	236	21.7	100.0	218	22.1	100.0	454	21.9	100.0

Table 2: Understanding questions: The coefficients are estimated through a Logit regression. P-values are reported in parentheses. The standard errors can be found in the Appendix. Columns 1 and 2: Right answer to the question on the understanding question on COVID-19 data. Columns 3 and : Right answer to question on Infection Z (hypothetical data). P-values are reported in parentheses. Standard errors for the same tables can be found in the Appendix. All coefficients for the control variables are reported.

	(1)	(2)	(3)	(4)
	Understanding Q.1: Real Data	Understanding Q.1: Real Data	Understanding Q.2: Hypothetical	Understanding Q.2: Hypothetical
main				
In Linear Group	2.021***	2.054***	4.634***	4.819***
	(0.000)	(0.000)	(0.000)	(0.000)
Confidence in Understanding Q.1		0.00886***		
		(0.000)		
Worry About Health Crisis		-0.0310		-0.0851
		(0.585)		(0.318)
COVID-19 News Checking		0.0780		0.0860
		(0.145)		(0.290)
Education		0.0213		0.152**
		(0.619)		(0.021)
Male		-0.147		0.321*
		(0.193)		(0.066)
Age		0.00445		0.0154**
		(0.268)		(0.012)
Democrat		0.00380		0.0870
		(0.977)		(0.660)
Republican		-0.0190		-0.183
		(0.895)		(0.413)
Confidence in Understanding Q.2				0.0308***
				(0.000)
Constant	-0.378***	-1.375***	-2.164***	-6.119***
	(0.000)	(0.001)	(0.000)	(0.000)
Observations	2074	1830	2074	1830

p-values in parentheses

* $p < 0.10$, ** $p < 0.05$, *** $p < 0.01$

Table 3: Determinants of making an accurate prediction (Columns 1 and 2) and an unreasonable prediction (Columns 3 and 4). The coefficients are estimated through Logit regressions. P-values are reported in parentheses. The standard errors can be found in the Appendix. All coefficients for the control variables are reported.

	(1) Accurate Prediction	(2) Accurate Prediction	(3) Unreasonable Prediction	(4) Unreasonable Prediction
main				
In Linear Group	0.489***	0.482***	-0.481***	-0.480***
	(0.000)	(0.000)	(0.000)	(0.000)
Confidence in Prediction		-0.00178		0.00188
		(0.447)		(0.411)
Worry About Health Crisis		-0.0112		0.0494
		(0.830)		(0.327)
COVID-19 News Checking		0.150***		-0.175***
		(0.002)		(0.000)
Education		0.0477		-0.0461
		(0.221)		(0.224)
Male		-0.0327		-0.0149
		(0.749)		(0.881)
Age		0.00182		-0.00480
		(0.616)		(0.175)
Democrat		0.0920		-0.106
		(0.437)		(0.360)
Republican		-0.181		0.221*
		(0.172)		(0.087)
Constant	-0.848***	-1.378***	0.585***	1.147***
	(0.000)	(0.000)	(0.000)	(0.001)
Observations	2074	1832	2074	1832

p-values in parentheses

* $p < 0.10$, ** $p < 0.05$, *** $p < 0.01$

Table 4: Determinants of worry about health crisis caused by Covid-19. The coefficients are estimated through ordered Logit regressions. P-values are reported in parentheses. Standard errors can be found in the Appendix. All coefficients for the control variables are reported.

	(1) Worry About Health Crisis	(2) Worry About Health Crisis	(3) Worry About Health Crisis
Worry About Health Crisis			
In Linear Group	0.141*	0.258*	0.327**
	(0.081)	(0.091)	(0.038)
COVID-19 News Checking		0.500***	0.434***
		(0.000)	(0.000)
Male		-0.806***	-0.654***
		(0.000)	(0.000)
Understanding Q.1: Real Data		-0.00425	0.00558
		(0.967)	(0.958)
Confidence in Understanding Q.1		-0.00134	-0.00152
		(0.706)	(0.674)
Understanding Q.2: Hypothetical		-0.137	-0.225
		(0.386)	(0.171)
Confidence in Understanding Q.2		-0.00374	-0.00428
		(0.302)	(0.246)
Accurate Prediction		0.156	0.218
		(0.404)	(0.255)
Unreasonable Prediction		0.225	0.325*
		(0.216)	(0.084)
Confidence in Prediction		0.00622***	0.00579***
		(0.005)	(0.009)
Democrat			0.732***
			(0.000)
Republican			-0.282**
			(0.017)
Worry About Economic Crisis			0.707***
			(0.000)
Live in city with <50K People			0.0156
			(0.880)
Live in city with >500K People			-0.132
			(0.280)
Education			-0.0258
			(0.473)
Age			-0.00132
			(0.694)
State of Residence			0.00777**
			(0.030)
Restrictions in the State			-0.156
			(0.160)
Observations	2074	1837	1828

p-values in parentheses

* $p < 0.10$, ** $p < 0.05$, *** $p < 0.01$

Table 5: Determinants for support for keeping shops closed (Columns 1-3) and suggested reopening day (Columns 4-6). Columns 1-3 report coefficients estimated through ordered Logit regressions and Columns 4-6 report coefficients obtained through ordinary least squares regressions (OLS). P-values are reported in parentheses. The standard errors can be found in the Appendix. All coefficients for the control variables are reported table

	(1)	(2)	(3)	(4)	(5)	(6)
	Support for	Support for	Support for	Days Until	Days Until	Days Until
Closing Businesses	Closing Businesses	Closing Businesses	Reopening Businesses	Reopening Businesses	Reopening Businesses	
main						
In Linear Group	0.0406	-0.378**	-0.424**	2.295	17.38**	14.65**
	(0.621)	(0.019)	(0.012)	(0.464)	(0.014)	(0.037)
Worry About Health Crisis		0.997***	1.067***		12.45***	13.14***
		(0.000)	(0.000)		(0.000)	(0.000)
COVID-19 News Checking		0.0288	0.0748		3.071*	3.932**
		(0.531)	(0.117)		(0.056)	(0.018)
Male		-0.112	-0.0890		10.53***	9.169***
		(0.242)	(0.366)		(0.002)	(0.006)
Understanding Q.1: Real Data		0.131	0.132		-1.236	-0.517
		(0.228)	(0.236)		(0.762)	(0.900)
Confidence in Understanding Q.1		0.00955***	0.00842**		0.109	0.0996
		(0.009)	(0.023)		(0.391)	(0.440)
Understanding Q.2: Hypothetical		0.300*	0.348**		-18.05**	-15.87**
		(0.075)	(0.047)		(0.012)	(0.026)
Confidence in Understanding Q.2		-0.000421	-0.000228		-0.310**	-0.299**
		(0.911)	(0.952)		(0.025)	(0.032)
Accurate Prediction		0.480**	0.450**		10.58*	9.343
		(0.012)	(0.019)		(0.093)	(0.138)
Unreasonable Prediction		0.0871	0.0806		6.590	4.787
		(0.635)	(0.665)		(0.277)	(0.431)
Confidence in Prediction		-0.00451*	-0.00426*		0.216***	0.205**
		(0.054)	(0.073)		(0.007)	(0.012)
Democrat			0.545***			0.107
			(0.000)			(0.977)
Republican			-0.491***			1.912
			(0.000)			(0.683)
Worry About Economic Crisis			-0.494***			-3.597*
			(0.000)			(0.069)
Live in city with <50K People			0.0314			6.259*
			(0.770)			(0.085)
Live in city with >500K People			0.0230			9.164**
			(0.858)			(0.037)
Education			-0.0258			-1.798
			(0.496)			(0.173)
Age			-0.00105			-0.151
			(0.769)			(0.192)
State of Residence			0.00274			-0.00686
			(0.456)			(0.957)
Restrictions in the State			-0.0175			-1.382
			(0.881)			(0.741)
In Linear Group						0
						(.)
Constant				65.38***	-0.312	24.09
				(0.000)	(0.979)	(0.155)
Observations	2074	1837	1828	2061	1828	1819

p-values in parentheses

* $p < 0.10$, ** $p < 0.05$, *** $p < 0.01$

Table 6: Determinants of likelihood to wear a mask when going out if provided with one (Columns 1-3) and supporting a tax to finance their distribution (Columns 4-6). The coefficients are estimated through ordered Logit regressions. P-values are reported in parentheses. The standard errors can be found in the Appendix. All coefficients for the control variables are reported. table

	(1) Likelihood to Wear Masks	(2) Likelihood to Wear Masks	(3) Likelihood to Wear Masks	(4) Support for Mask-Buying Tax	(5) Support for Mask-Buying Tax	(6) Support for Mask-Buying Tax
main						
In Linear Group	0.00311	-0.314**	-0.350**	-0.0218	0.307**	0.305**
	(0.970)	(0.045)	(0.029)	(0.780)	(0.042)	(0.046)
Worry About Health Crisis		0.907***	0.908***		0.481***	0.471***
		(0.000)	(0.000)		(0.000)	(0.000)
COVID-19 News Checking		0.138***	0.129***		0.0403	0.0682
		(0.003)	(0.006)		(0.341)	(0.116)
Male		-0.255***	-0.270***		0.0372	0.0455
		(0.007)	(0.005)		(0.673)	(0.612)
Understanding Q.1: Real Data		0.0281	0.0136		0.152	0.169*
		(0.796)	(0.902)		(0.133)	(0.097)
Confidence in Understanding Q.1		0.00571	0.00493		0.00648*	0.00602*
		(0.125)	(0.192)		(0.065)	(0.088)
Understanding Q.2: Hypothetical		0.189	0.237		-0.454***	-0.452***
		(0.249)	(0.157)		(0.004)	(0.004)
Confidence in Understanding Q.2		0.00250	0.00272		-0.0108***	-0.0112***
		(0.510)	(0.479)		(0.003)	(0.002)
Accurate Prediction		0.435**	0.431**		0.186	0.141
		(0.020)	(0.022)		(0.312)	(0.444)
Unreasonable Prediction		0.497***	0.493***		0.165	0.147
		(0.007)	(0.007)		(0.357)	(0.414)
Confidence in Prediction		0.00211	0.00276		0.00675***	0.00734***
		(0.352)	(0.231)		(0.002)	(0.001)
Democrat			0.161			0.378***
			(0.154)			(0.000)
Republican			-0.384***			-0.261**
			(0.001)			(0.024)
Worry About Economic Crisis			-0.132**			-0.0979*
			(0.021)			(0.069)
Live in city with <50K People			0.0832			0.115
			(0.424)			(0.240)
Live in city with >500K People			0.588***			0.0488
			(0.000)			(0.681)
Education			-0.0767**			-0.0209
			(0.040)			(0.543)
Age			0.00713**			-0.00942***
			(0.041)			(0.004)
State of Residence			0.0170***			-0.00313
			(0.000)			(0.358)
Restrictions in the State			-0.154			-0.122
			(0.177)			(0.258)
Likelihood to Wear Masks					0.648***	0.617***
					(0.000)	(0.000)
Observations	2072	1835	1826	2072	1834	1825

p-values in parentheses

* $p < 0.10$, ** $p < 0.05$, *** $p < 0.01$

Table 7: The table reports Odds Ratios for Logit Regressions: Worry About Health Crisis, Likelihood to Wear Masks, Support for Mask-Buying Tax, Support for Closing Businesses, Understanding Q.1: Real Data, Understanding Q.2: Hypothetical, Accurate Prediction, Unreasonable Prediction. The controls used in each of these regression is the same as in the last column of each regression in Tables 2-6.

	(1) Worry About Health Crisis	(2) Likelihood to Wear Masks	(3) Support for Mask-Buying Tax	(4) Support for Closing Businesses	(5) Understanding Q.1: Real Data	(6) Understanding Q.2: Hypothetical	(7) Accurate Prediction	(8) Unreasonable Prediction
main								
In Linear Group	1.387*	0.705*	1.356*	0.654*	7.800***	123.9***	1.619***	0.619***
	(0.218)	(0.113)	(0.207)	(0.110)	(0.902)	(23.13)	(0.159)	(0.0594)
COVID-19 News Checking	1.543***	1.138**	1.071	1.078	1.081	1.090	1.162**	0.840***
	(0.0718)	(0.0537)	(0.0464)	(0.0514)	(0.0578)	(0.0886)	(0.0563)	(0.0398)
Male	0.520***	0.763**	1.047	0.915	0.864	1.379	0.968	0.985
	(0.0486)	(0.0735)	(0.0937)	(0.0900)	(0.0972)	(0.241)	(0.0988)	(0.0980)
Understanding Q.1: Real Data	1.006	1.014	1.184	1.141				
	(0.107)	(0.112)	(0.120)	(0.127)				
Confidence in Understanding Q.1	0.998	1.005	1.006	1.008*	1.009***			
	(0.00361)	(0.00379)	(0.00355)	(0.00375)	(0.00253)			
Understanding Q.2: Hypothetical	0.799	1.267	0.636**	1.416*				
	(0.131)	(0.212)	(0.101)	(0.247)				
Confidence in Understanding Q.2	0.996	1.003	0.989**	1.000		1.031***		
	(0.00368)	(0.00385)	(0.00360)	(0.00379)		(0.00424)		
Accurate Prediction	1.244	1.539*	1.152	1.569*				
	(0.238)	(0.290)	(0.213)	(0.302)				
Unreasonable Prediction	1.384	1.638**	1.159	1.084				
	(0.260)	(0.301)	(0.209)	(0.202)				
Confidence in Prediction	1.006**	1.003	1.007***	0.996			0.998	1.002
	(0.00225)	(0.00231)	(0.00221)	(0.00236)			(0.00234)	(0.00229)
Democrat	2.080***	1.175	1.459***	1.725***	1.004	1.091	1.096	0.900
	(0.225)	(0.133)	(0.152)	(0.200)	(0.131)	(0.216)	(0.130)	(0.104)
Republican	0.754*	0.681**	0.770*	0.612***	0.981	0.833	0.834	1.247
	(0.0893)	(0.0822)	(0.0891)	(0.0735)	(0.141)	(0.186)	(0.111)	(0.161)
Worry About Economic Crisis	2.028***	0.876*	0.907	0.610***				
	(0.112)	(0.0502)	(0.0488)	(0.0374)				
Live in city with ¡50K People	1.016	1.087	1.122	1.032				
	(0.105)	(0.113)	(0.110)	(0.111)				
Live in city with ¿500K People	0.876	1.801***	1.050	1.023				
	(0.107)	(0.233)	(0.124)	(0.132)				
Education	0.975	0.926*	0.979	0.975	1.022	1.164*	1.049	0.955
	(0.0350)	(0.0347)	(0.0338)	(0.0369)	(0.0438)	(0.0768)	(0.0409)	(0.0362)
Age	0.999	1.007*	0.991**	0.999	1.004	1.016*	1.002	0.995
	(0.00336)	(0.00352)	(0.00322)	(0.00355)	(0.00403)	(0.00624)	(0.00363)	(0.00352)
State of Residence	1.008*	1.017***	0.997	1.003				
	(0.00362)	(0.00402)	(0.00339)	(0.00368)				
Restrictions in the State	0.855	0.857	0.885	0.983				
	(0.0951)	(0.0978)	(0.0957)	(0.115)				
Worry About Health Crisis		2.480***	1.602***	2.907***	0.969	0.918	0.989	1.051
		(0.136)	(0.0862)	(0.165)	(0.0550)	(0.0782)	(0.0513)	(0.0530)
Likelihood to Wear Masks			1.854***					
			(0.0935)					
Observations	1828	1826	1825	1828	1830	1830	1832	1832

Exponentiated coefficients; Standard errors in parentheses

* $p < 0.05$, ** $p < 0.01$, *** $p < 0.001$

References

Benvenuto, D. et al. (2020). Application of the arima model on the covid-2019 epidemic dataset. Data in Brief 29(105340), 1–4.

Brzezinski, A. et al. (2020). Belief in science influences physical distancing in response to covid-19 lockdown policies. University of Chicago, Becker Friedman Institute for Economics Working Paper 2020-56.

Bursztyn, L. et al. (2020). Misinformation during a pandemic. University of Chicago, Becker Friedman Institute for Economics Working Paper No. 2020-44.

Everett, J. et al. (2020). The effectiveness of moral messages on public health behavioral intentions during the covid-19 pandemic. PsyArXiv Preprints.

FinancialTimes (2020). Coronavirus tracked: has the epidemic peaked near you? Financial TImes.

Garfin, D. R. et al. (2020). The novel coronavirus (covid-2019) outbreak: Amplification of public health consequences by media exposure. Health Psychology 5(39), 355–357.

Gladen, B. C. . W. J. R. (1983). Brief reports on graphing rate rations. American Journal of Epidemiology.

Guardian, T. (2020). How coronavirus spread across the globe - visualised. The Guardian.

Heckler, A. F. et al. (2013). Student accuracy in reading logarithmic plots: the problem and how to fix it. IEEE Frontiers in Education Conference.

Kosslyn, S. (2006). Graph design for the eye and mind. Oxford University Press.

Levine, M. A. et al. (2010). Relative risk and odds ratio data are still portrayed with inappropriate scales in the medical literature. Journal of Clinical Epidemiology, 1045–1047.

Menge, D. et al. (2018). Logarithmic scales in ecological data presentation may cause misinterpretation. Nature Ecology & Evolution 9(2), 1393–1402.

NewYorkTimes (2020a). Coronavirus deaths by u.s. state and country over time: Daily tracker. New York Times.

NewYorkTimes (2020b). A different way to chart the spread of coronavirus. New York Times.

Okan, Y. et al. (2016). How people with low and high graph literacy process health graphs: Evidence from eye-tracking. Journal of Behavioral Decision Making 29.2-3, 271–294.

Phlol, N. and B. Musil (2020). Modeling compliance with covid-19 prevention guidelines: The critical role of trust in science. PsyArXiv Preprints.

Pidgeon, N. and B. Fischhoff (2011). The role of social and decision sciences in communicating uncertain climate risks. Nature Climate Change, 35–41.

Ryan, W. and E. Evers (2020). ”logarithmic axis graphs distort lay judgment. PsyArXiv Preprints.

Sevi, S. et al. (2020). Logarithmic versus linear visualizations of covid-19 cases do not affect citizens' support for confinement. Canadian Journal of Political Science, 1–6.

TheWashingtonPost (2020). What does exponential growth mean in the context of covid-19? The

Washington Post.

Van Bavel, J. et al. (2020). Using social and behavioural science to support covid-19 pandemic response. PsyArXiv Preprints.

Vox (2020). Trump's coronavirus death toll estimate exposes his failure. Vox.
