# Supplementary material for: The scale of COVID‐19 graphs affects understanding, attitudes, and policy preferences
Source: Health Econ. 2020 Aug 25;29(11):1482–94. doi: 10.1002/hec.4143 (PMC7461444; doi:10.1002/hec.4143)
Supplement: Supplementary file 3 — Supplementary Material [file HEC-29-1482-s003.docx]

Reviewer 1:

Thank you for the opportunity to read this very interesting, and highly topical and relevant manuscript. I think it is very good and could add to the current literature on public understanding of data. On the whole the paper is very good and easy to read.

*We thank the referee for the kind words and for taking the time to review our manuscript. We really appreciated the comments and tried to address them to the best of our ability.*

The Tables, however, need more coherent and intuitive names/titles and variable labels. My more specific comments are below. My comments: Structured by order they appear in the manuscript

In the Abstract, line 9, you state ‘Reducing misinformation…’ This is not technically ‘misinformation, just an alternative way of expressing the data.

*We agree with the referee and following this suggestion we changed the wording from “Reducing misinformation” to “Providing the public with information in ways they understand better” (in the abstract) and “Providing the public with clear information” (in the text).*

*We also changed the title from “Covid-19 Data: The Logarithmic Scale Misinforms the Public and Affects Policy Preferences” because it used the word “misinform” that, as correctly noted by the referee, is imprecise. The title is now “The Scale of COVID-19 Graphs Affects Understanding, Attitudes, and Policy Preferences”*

I could not see the Supplementary Material Appendix containing the questions and their order. I would quite like to look at this.

*We have now included a table with all the questions in the Appendix, which is included in the resubmission. Moreover, we include the Questionnaire here as well to avoid problems (the same is in the Appendix).*

I suggest you split Section 2 into two: one explaining the experiment and one explaining the results (from ‘Health and Economic Crises’ onwards).

*In line with the suggestion of the referee, we now have a section that explains the experiment and one that presents the results. We do not include the screenshots of the changes as we restructured these sections entirely.*

I assume tables 2-9 are for an Appendix? As such, perhaps you could label them “Table A#” etc., so it is obvious they are for an Appendix.

*As we restructured the articles, we also changed the Tables. In the article, we now have the following tables:*

1) *Demographics: describing the demographic characteristic of our sample*
2) *Understanding: showing that the linear group has a better understanding of the graphs on COVID-19 and on Infection-Z*
3) *Prediction: showing that the linear group makes better predictions on the evolution of the pandemic*
4) *Shops closed and reopening day: showing the difference between the two groups in terms of preferences for keeping non-essential business closed and of when to reopen them*
5) *Tax and masks: showing the difference between the two groups in terms of support for a tax to provide masks, and on mask use.*

*In the appendix we include the tables that were previously in the text, with all the robustness checks. As suggested by the referee they are now labeled as Table A#.*

Also, these tables need to be tidied up (such as making more intuitive table names and adding labels to the variables etc., but I assume this can be done at a later stage).

*As noted in response to the previous comment, we tried to improve our tables. We have included clearer descriptions and relabeled the variables to make them easier to understand. We have also introduced longer captions.*

Presenting Logit and Probit results is a bit ‘over kill’ and I would suggest picking one (looks like you prefer Logit) and using that and the OLS as a comparison. However the editor may think it is fine to keep all nine columns.

*In line with the suggestion of the referee we only include the ordered logit. To allow referees and other researchers to understand the results more easily we have included also a table with odds ratios for all the coefficients. We have moved all other tables to the appendix as a robustness check of our results.*

Also in Tables 2-9, are the logit/probit results coefficients or marginal effects or odds ratios? This could be made more explicit.

*We agree with the referee that the structure was not sufficiently clear. The tables shown in the first version of the paper (and in the current one) show the coefficients from the (ordered) logit regressions. However, interpreting these coefficients might result tedious, so we have included a table with odds ratios. Hopefully, this will allow readers to interpret our results more easily, while still leaving the possibility to check the robustness of our results. In line with the referee’s suggestions we have also included a description of the tables listing what results are being presented.*

Include standard errors not p-values? Again, the editor may think it is OK to include p-values not standard errors.

*We agree with the referee that showing standard errors is important. For this reason, we have included them in the tables in the appendix, whereas we kept the p-values in the text to allow for an easier reading of the results and their significance. We are happy to modify this according to* the referees’/editors’ preferences if they believe that other solutions would be better

The “Summary Statistics” table needs a title and Table number.

*We thank the referee for spotting this! We have now included a title and a table number*

Also, the variables need labelling in a intuitive way.

*We have now relabeled all variables in a way that is more intuitive to read and included descriptions for the variables in each of the tables, including the scale used to measure them*.

Are the differences between groups statistically significant? Perhaps you could report either (i) paired t-tests or (ii) coefficients of a regression where you use those variables to predict group membership.

*We have run both checks suggested by the referee. We have now included a demographics table showing the composition of both groups. Below we present a regression assessing whether demographics can predict the group where the participant belongs. We agree with the referee that demographics should not be a good predictor of the group the participant was assigned to. We are happy to report that indeed the demographic variables are not good predictors, which shows that the randomization seems to have created balanced groups. Of course, this is true only with respect to observable variables. Results are reported below:*

To make the paper easier to read, I would drop mention of “Group A” and refer to people exposed to the linear scale. This saves the reader from having to remember what each group is.

*We agree with the referee and we replaced “Group A”/”Group B” with “Linear Group” and “Log Group” throughout the text.*

Your discussion section seems to add more results and succinctly summarise the earlier results into one table.

*We agree that the old structure was a bit confusing. Therefore, as noted above, we restructured Section II entirely. We have a section “Experiment” and one for “Results and Discussion”. We hope this addresses the concern of the referee.*

I think Table 1 should stay in the main text and Tables 2-9 go in an Appendix (see above and below). That said, I think Table 1 should include the coefficients from all Tables (2-9), and not just selected ones.

*We agree with the referee that the structure was not sufficiently clear. As noted above, we changed most of the tables to make them easier to understand. Table 1 should easily show the impact of being in one group (the linear) with respect to being in the other (the log one) without having to work out odds ratio manually. For all the regressions we show all the coefficients. This table conveys no new information, but we hope that it helps the reader understanding our results. We are fine with deleting it if the referee think we shouldn’t have it.*

. Table 1 also needs clarifying. What is it measuring/showing? I think it is column 3 of tables 2-9. Some of the numbers don’t marry up to the respective tables in the text T2-T9, see below:

Odds ratio is also wrong. Also, in the last row in Table 1, the relationship between the odds ratio and the coefficient is wrong. We know coeff = ln(OR), but this is not true in the last row. So I think something has gone wrong here.

*We thank the referee for spotting this! The sets of controls weren’t matching between the regressions and the table, so the odds ratios did not match the coefficients in the tables reported. We apologize for this and have included new tables. We also made sure to include all regressors in the odds ratio table.*

Reviewer: 2

Comments to the Author

This paper examines the impact of viewing cumulative death data presented on linear versus logarithmic scales on policy preferences, level of worry about the pandemic-induced health and economic crises, and future predictions of deaths. These findings that exposure to data using different scales can alter these behavioral preferences is interesting and are policy relevant. The paper, however, could use major restructuring in order to present their findings in a more linear and understandable manner. Additionally, the authors have some opposing findings that may be due to underlying behavioral characteristics not accounted for in their study. My comments below are mainly focused on 1) restructuring and clarification and 2) experimental design.

*We thank the referee for the kind words and for taking the time to review our manuscript. We really appreciated the comments and tried to address them to the best of our ability.*

1. In the first paragraph on page 2, it would be worthwhile to add a line as to why some media sources choose to present information on a linear versus a log scale in the first place.

*We agree with the referee’s suggestion. We included a paragraph mentioning the New York Times’ stated rationale for using logarithmic scale graphs, as well as a few citations in epidemiology and data visualization literature that suggest using logarithmic scales for this kind of exponential growth.*

2. I am not convinced about the policy recommendation to show both scales as this may likely cause even more confusion than showing one or the other (page 2, line 30).

*We agree with the referee that showing both scales might confuse the reader. We changed that part of the policy recommendation as shown in the screenshot below.*

3. It would be useful to include more information on the sample of participants for this experiment (e.g. are they representative of the US? Did you collect any additional information on them besides their gender?). The authors may be suffering from sample selection bias and/or other heterogeneities that may be why some findings are opposing.

*We agree with the referee. We added a table that describes the characteristics of the overall sample and of the two groups that we include here as well as in the manuscript.*

*While our sample is not representative of the US population, there are no statistically significant differences between the characteristics of the groups. We tested this in two different ways: 1) a pairwise comparison for demographics in the two groups and 2) a regression attempting to predict where the participant would belong based on demographics. Neither showed any significance (See table below).*

4. It would be good if the authors mentioned how they measure respondents’ level of worry (page 3, line 33).

*Following the referee’s suggestion, we now specify in the article that we measure the level of worry using a five points Likert scale from "not worried at all" to "extremely worried". The full question, like all the other questions, is also in the appendix.*

5. Section 2 could benefit from first explaining the experiment and types of questions asked to both groups, and then followed by all the results rather than the current mixture of methods and results.

*We thank the referee for this suggestion, which we believe can increase the clarity of the paper. We rearranged the sections as suggested by the referee. We now have a section that explains the experiment and one that presents the results. We do not include screenshots and we do not mark the sections in red as they are almost completely restructured.*

6. The authors state that the linear graph group is more worried about the health crisis but not the economic crisis (page 3, line 37). It is likely that since these graphs do not show anything about the economy (like a graph of unemployment level might for example), that they really can’t tell you anything about how worried people are about the economic crisis. So I am not sure how meaningful is this measure of worry about the economic crisis.

*We agree with the referee that the graphs do not show anything about the economy, and therefore it is reasonable that the scale adopted in these graphs does not directly affect worry about the economic crisis. Nevertheless, since the economic crisis is strictly connected with the health crisis (e.g. more deaths might imply a longer or more strict lockdown, and hence a more severe economic crisis), we thought it was interesting to also ask participants their worry about the economic crisis. We had included the table to avoid “hiding” a non-result. However, given the concern raised by the referee, we eliminated the table on economic worries from the article (but we keep it in the appendix for transparency). For transparency, we also have a sentence stating that we do not find an effect on economic worry.*

7. It does not seem like this experiment has a control group, so I would caution mentioning anything about causality (page 3, line 51).

*The referee is correct and we do not have a control group. We made sure to adjust the text accordingly.*

8. This sentence that people in group A are less in favor of closing stores but want to keep stores closed for longer (page 3, line 59) should be clarified since as it stands, it does not make much sense. In the discussion, the authors explain that in fact group A participants do not want stores to be closed to begin with but now that they are closed, they want them closed for longer. That clarification here would be appreciated.

*We acknowledge that these results aren’t what one would expect. In fact, when we* [*pre-registered*](https://osf.io/ehk3y/) *the experiment we hypothesized that the group who supported more strongly closing stores would also prefer that they stayed closed for longer. But this is not what we observe.*

*We attempted to explain our result more clearly in the text, and we hope that the referee finds this explanation convincing. However, we acknowledge - here and in the article - that this is merely a conjecture. Independently from the reasons behind the findings, we believe that what is important is that the scale of the graph can alter attitudes and preferences.*

9. It would be good to distinguish whether all of the authors’ findings are for participants in group A, group B, or both.

*In the new version, we tried to address the comment of the referee by always specifying the group we refer to. There should be no instances in which this is not specified. But we are happy to address this in case we missed something.*

10. The authors’ first understanding question (page 4, line 37), it is unclear to me how this checks understanding of log versus linear graphs. Both graphs increase over the time period, so if the participant answers yes, I am not sure it means that they understand the difference between a log versus a linear graph.

*We apologize if the description of the question was unclear. In the first understanding question, we show the two groups the graph on the evolution of COVID-19 related deaths in the scale to which they were assigned. We then ask them whether they believe there were more new deaths between March 31st and April 6th or between April 6th and April 12th (“did the total number of deaths increase MORE between March 31st and April 6th…”; the word “MORE” was written in capital letters in the questionnaire; respondents could also answer that the number of new deaths were the same or “I don’t know”). The correct answer is that there were more new deaths between April 6th and April 12th, and this answer is evident from either graph if one reads it correctly. However, most of the people in the log group failed to identify that there were more new deaths between April 6th and April 12th than between March 31st and April 6th . This is some evidence that they didn’t understand how to read the log scale graph.*

11. It would be good to make clear that the prediction of April 25th deaths made by participants (page 4, line 55) is for cumulative deaths and not deaths on April 25th as this would not match the ARIMA-forecasted estimate.

*In line with the suggestion of the referee we changed the wording to reflect that we are referring to cumulative deaths. We had specified this clearly in the questionnaire.*

12. What are the training start and end dates for the ARIMA model used by the authors? If it is since the beginning of the pandemic, then the model may not be representative of the exponential increase in deaths starting around March 25th.

*The training date used is February 15^th^ 2020, which is the same date from which the graphs start. The prediction of the ARIMA of 55,791 is pretty similar to the observed number of 54,256, so we believe our forecasting to be reasonable. While we’d be open to use different start dates, we fear that since we did not specify a starting date in the* [*pre-registration*](https://osf.io/ehk3y/)*, any choice different from the beginning of the graph might look arbitrary in the eye of a reader. Moreover, since we show respondents the entire graph, by using a different starting date we would be giving the ARIMA and respondents different information sets. This would complicate the interpretation of the results.*

*However, we understand that the point raised by the referee is important and that the results should hold when using a different starting date. Therefore, we set the start date one week later and replicate the analysis. Here we report the results. We note that the prediction is extremely similar to that of the ARIMA we used. The ARIMA with a later starting date predicts 55,788 COVID-19 related deaths instead of 55,791 (a difference of only 3 deaths). To check the robustness of our results we re-ran the analysis with the new CIs. Both the sign and the statistical significance of the findings remain the same.*

*In any case, we remark that we are not expecting respondents to make accurate predictions. We only consider unreasonable predictions those that are outside of the 99% CI. The upper and lower bounds of this CI were 46,895.47 to 64,685.95. The number of deaths on the day of the survey was 39,014. Therefore, the observed increase in the number of COVID-19 the week considered was 16,777. To make an unreasonably high prediction, one should have predicted a number of deaths of at least 25,672. This is an error of almost 9,000 deaths (or 53% more than the actual increase). To make an unreasonably low prediction, one should have predicted a number of deaths of at most 7,881, which is less than 50% of the actual increase. These are very large margin, thus meaning that who made an unreasonable prediction was really far off.*

13. It would be interesting to see whether the predictions made by participants in group B fall within the reasonable range for a forecast using the logarithmic data. It may be possible that the participants in group B are not necessarily confused, but rather are simply anchored on the log scale.

*We used R's auto.arima command to find out the best possible fit for the data. Therefore, the parameters p, d and q for ARIMA(p,d,q) were found through simulations to yield the best possible set of parameters to make forecasting. While no forecast can be perfect, we don't expect participants to make perfect predictions either. In fact, we consider as "accurate" all those falling in the 95% confidence interval, which is fairly wide in this case. We further wish to note that one of the advantages in using ARIMA rather than fitting a curve on a graph is to avoid fitting on one scale as the model runs over raw data. We acknowledge that it would be interesting to try different forecasting methods, maybe based on different logics, but we stated which method we were going to use in the pre-registration and we feel that ex post changes in analysis could be problematic. In the pre-registration we opted for ARIMA because it is widely used, also to forecast the evolution of COVID-19.*

14. The authors have seemingly opposing findings of those in group A who want businesses to stay closed by also did not want them closed in the first place. The exponential increase in deaths shown on the linear scale does not necessarily explain why these participants did not want businesses closed in the first place. Is it possible that there may have been other underlying behavioral factors (e.g. time preferences, risk preferences, etc) not specified in this study that may tease out any heterogeneities in the responses in group A?

*As noted in response to point 8, we agree with the referee that the results on this point are not what one would expect. In fact, we did pre-register a hypothesis that is not in line with this specific result. One preliminary clarification we would like to make is that the Linear Group (former Group A) does want to close stores (4.09 on a Likert scale from 1 to 5). But when we include the relevant controls, they support closing stores less than the Log Group. However, this is in relative terms only. If we understood correctly the comment from referee one, the referees could not see the Supplementary Material Appendix where this data was reported. We apologize for this, and we hope that this clarifies the misunderstanding.*

*Said this, the tests we carried out reveal that there are no statistically significant differences in terms of demographic characteristics between the two groups. Thus, it seems that the randomization has created balanced groups. However, the referee is correct in noting that this balance only exists on our observable variables. Therefore, we cannot rule out other differences that might affect preferences and might be heterogeneously distributed across the two groups. To conclude, we note that the groups do not present statistically significant differences in terms of characteristics that generally affect risk and time preferences (e.g. age and gender).*
